# A postnatal network of co-hepato/pancreatic stem/progenitors in the biliary trees of pigs and humans

**DOI:** 10.1038/s41536-023-00303-5

**Published:** 2023-08-01

**Authors:** Wencheng Zhang, Xicheng Wang, Giacomo Lanzoni, Eliane Wauthier, Sean Simpson, Jennifer Ashley Ezzell, Amanda Allen, Carolyn Suitt, Jonah Krolik, Alexander Jhirad, Juan Dominguez-Bendala, Vincenzo Cardinale, Domenico Alvaro, Diletta Overi, Eugenio Gaudio, Praveen Sethupathy, Guido Carpino, Christopher Adin, Jorge A Piedrahita, Kyle Mathews, Zhiying He, Lola McAdams Reid

**Affiliations:** 1 Department of Cell Biology and Physiology, University of North Carolina (UNC) School of Medicine, Chapel Hill, NC 27599 USA; 2 Institute for Regenerative Medicine, Shanghai East Hospital, School of Life Sciences and Technology, Tongji University School of Medicine, 200123 Shanghai, China; 3 Shanghai Engineering Research Center of Stem Cells Translational Medicine, 200335 Shanghai, China; 4 Shanghai Institute of Stem Cell Research and Clinical Translation, 200120 Shanghai, China; 5 Diabetes Research Institute, Leonard Miller School of Medicine, 1450 N.W. 10th Avenue, Miami, FL 33136 USA; 6 Department of Molecular Biomedical Sciences, North Carolina State University (NCSU) College of Veterinary Medicine, Raleigh, NC 27606 USA; 7 Comparative Medicine Institute, NCSU, Raleigh, NC 27606 USA; 8 Center for Gastrointestinal Biology and Disease (CGIBD), UNC School of Medicine, Chapel Hill, NC 27599 USA; 9 Department of Medico-Surgical Sciences and Biotechnologies, Sapienza University, Rome, Latina 04100 Italy; 10 Department of Translational and Precision Medicine, Sapienza University, Rome, 00185 Italy; 11 Department of Anatomical, Histological, Forensic Medicine and Orthopedics Sciences, Sapienza University, Rome, 00161 Italy; 12 Department of Biomedical Sciences, Cornell University College of Veterinary Medicine, Ithaca, NY 14853 USA; 13 Department of Clinical Sciences, Soft Tissue and Oncologic Surgery Service, College of Veterinary Medicine, NCSU, Raleigh, NC 27606 USA; 14 Program in Molecular Biology and Biotechnology, UNC School of Medicine, Chapel Hill, NC 27599 USA; 15 Present Address: Department of Small Animal Clinical Sciences, University of Florida College of Veterinary Medicine, Gainesville, FL 32608 USA

**Keywords:** Regeneration, Adult stem cells

## Abstract

A network of co-hepato/pancreatic stem/progenitors exists in pigs and humans in Brunner’s Glands in the submucosa of the duodenum, in peribiliary glands (PBGs) of intrahepatic and extrahepatic biliary trees, and in pancreatic duct glands (PDGs) of intrapancreatic biliary trees, collectively supporting hepatic and pancreatic regeneration postnatally. The network is found in humans postnatally throughout life and, so far, has been demonstrated in pigs postnatally at least through to young adulthood. These stem/progenitors in vivo in pigs are in highest numbers in Brunner’s Glands and in PDGs nearest the duodenum, and in humans are in Brunner’s Glands and in PBGs in the hepato/pancreatic common duct, a duct missing postnatally in pigs. Elsewhere in PDGs in pigs and in all PDGs in humans are only committed unipotent or bipotent progenitors. Stem/progenitors have genetic signatures in liver/pancreas-related RNA-seq data based on correlation, hierarchical clustering, differential gene expression and principal component analyses (PCA). Gene expression includes representative traits of pluripotency genes (SOX2, OCT4), endodermal transcription factors (e.g. SOX9, SOX17, PDX1), other stem cell traits (e.g. NCAM, CD44, sodium iodide symporter or NIS), and proliferation biomarkers (Ki67). Hepato/pancreatic multipotentiality was demonstrated by the stem/progenitors’ responses under distinct ex vivo conditions or in vivo when patch grafted as organoids onto the liver versus the pancreas. Therefore, pigs are logical hosts for translational/preclinical studies for cell therapies with these stem/progenitors for hepatic and pancreatic dysfunctions.

## Introduction

The hepato/pancreatic biliary tree in all mammals comprises ramifying ducts connecting the liver and pancreas to the duodenum^1–5^. The biliary tree, its connections to the duodenum in all domestic animals^2,4,6–8^ and in humans^9–13^, and its embryological development have been described previously^1,6,8,14–17^.

Various breeds of pigs (*Sus scrofa domestics*) are major animal species used in translational research, surgical models, and procedural training and are used increasingly as alternatives to primates for preclinical testing of pharmaceuticals and of bioengineered products^18–20^. The similarities between pigs and humans in their anatomic and physiologic characteristics in organs have made them a primary species of interest as organ donors for xenograft procedures^21–23^.

Regulatory agencies require that a drug or treatment be proven safe and effective in two species: a small animal (mice, rats) and a large one (pigs, primates). After a review of the biological properties of liver, biliary tree, pancreas, and duodenum in various large animals, we have chosen pigs as models for preclinical studies of stem/progenitor cell therapies for their ready availability, their costs, and especially their many features that parallel those in humans. The veterinary literature provides extensive characterizations in all domestic animal species of the biliary tree and its connections to the duodenum^3,24–28^. However, there is no information on whether pigs are like humans and postnatally have a network of hepato/pancreatic co-stem/progenitor populations^2,5–8,29–31^. Therefore, it was necessary to determine if they exist and, if so, to characterize the cells and the network with respect to the cells’ genetic signatures, in situ locations, and behavior ex vivo and when transplanted in vivo.

Extensive characterizations of the human network have offered guides of isolation and management, to the candidate phenotypic traits of the hepato/pancreatic co-stem/progenitors and of the network^5,26,31–35^. In humans, the complex network is comprised of stem cells in extramural PBGs (tethered to the outside of large ducts that are ≥300 μm), at the bottoms (near the bile duct centers) of intramural PBGs (within the duct walls), and in duodenal glands, also called Brunner’s Glands, in the submucosa of the duodenum^5,29–33,36^. The extramural PBGs^30,32^ and Brunner’s Glands^34,36^ contain the most primitive of the stem cell populations identified and have anatomical connections to the intramural population^5,32^. Key known functions of the biliary tree and pancreatic ducts are as a conduit for bile from the liver and for digestive enzymes from the pancreas, and, discovered within the last decade, as a reservoir of multiple stem/progenitor subpopulations contributing throughout life to biliary, hepatic and pancreatic regenerative processes both in quiescent states and in diseased ones^5,29–31,37,38^.

Intramural PBGs are found throughout the intrahepatic and extrahepatic biliary tree, especially at the branching points, in the cystic duct, with the highest numbers in the hepato-pancreatic common duct and the second highest numbers in the large intrahepatic bile ducts^5,30,32,39^. Mapping the phenotypic traits of the subpopulations of stem/progenitors in the intramural niches in humans has made apparent a radial axis of maturation from a deep position within the walls of the bile ducts. The intramural PBGs have sites at positions near the fibromuscular layer (center of the bile duct walls) and at which the epithelial cells are the most primitive; maturation of cells occurs with the transition of cells with increasing proximity to the duct’s lumen, at which site the cells have matured. In summary, the sites at the centers of the bile duct walls are presumptive stem cell crypts, yielding daughter cells undergoing maturation to adult fates when cells reach the duct’s lumen.

There is also a proximal to distal axis of maturation from the duodenum via the extrahepatic and intrahepatic (or intrapancreatic) biliary tree to the organ (liver or pancreas) in which the radial axes of lineages in ducts near to the liver contain cells that mature to have hepatic traits versus those in ducts near to or in the pancreas, acquire pancreatic traits. Therefore, the regenerative processes for the liver and the pancreas are ongoing throughout life via the maturation of cells from stem cell crypts throughout the biliary tree^5,31,32,36,39^.

In humans, the intramural stem/progenitors in the PBGs give rise to late-stage stem/progenitors located at the bottoms of villi in the gallbladder that has no PBGs^33^ and to hepatic stem cells (HpSCs) and then to hepatoblasts (HBs) in or near the canals of Hering^40–43^. The HpSCs and HBs give rise to mature hepatic parenchymal cells (hepatocytes and cholangiocytes) that continue the stepwise maturational progression ending within the liver acini with terminally differentiated, polyploid parenchymal cells located near the central veins^40,44,45^. The terminal-Axin 2^+^ hepatocytes, linked on their lateral borders to endothelia forming the central vein, are diploid and are hypothesized to serve a regenerative role replacing senescing, polyploid hepatocytes^46–48^. The contributions of the terminal, diploid hepatocytes to facets of liver regeneration in rats have been implicated further by a recent extensive study of transcriptomic analyses characterizing progenitor and mature traits in parenchymal cells of different ploidy and in particular zones of the liver acini. Although such a comprehensive and extensive transcriptomic study has yet to be done in pigs, the implications are that the regenerative contributions of the terminal, diploid parenchyma, located by the central vein, handle apoptotic cells in normal liver and perhaps also certain pathologies such as damage from zone 3 toxins (e.g. carbon tetrachloride, CCl4). These regenerative responses complement those contributed by multipotent co-hepato/pancreatic stem cells within the biliary tree.

Although in humans there are stem cells in the accessory duct to the dorsal pancreas, there are only bipotent and unipotent progenitors within the pancreas proper; rare cells within the pancreas express pluripotency genes, and in those cells, the expression is primarily or entirely cytoplasmic^49,50^. The progression of maturational lineage stages within most regions of the pancreas has yet to be defined in either humans or pigs.

Stem/progenitors have been found to contribute to liver and pancreatic organogenesis throughout life in humans^5,30,31^. The network responds dramatically to regenerative demands as indicated in mice^51^ and in humans^5,29^ and now also in pigs. In addition, the co-hepato/pancreatic stem/progenitor network is recognized now as central to an understanding of certain pathologies relevant to either liver or pancreas or both^29,31,52,53^.

Here we demonstrate parallels in the networks of co-hepato/pancreatic stem/progenitors in pigs and humans. We used genetic signature studies by which to identify key biomarkers and then used those to locate the niches of these cells within the liver, biliary tree, duodenum, and pancreas in pigs. Proof of their multipotentiality was achieved by isolating the stem/progenitors and assessing their ability to give rise to cells with hepatic as well as pancreatic fates ex vivo under wholly defined culture conditions or with transplantation in vivo using patch grafting strategies for transplantation of organoids into solid organs such as liver and pancreas^54,55^.

## Results

### Gross anatomy of the biliary tree and pancreatic duct system in pigs

The gross anatomy of the hepato/pancreatic biliary tree is similar in all mammalian species in being comprised of ramifying ducts within the liver and the pancreas plus branching regions that are exterior to the organs and connecting the liver to the duodenum via the common bile duct and connecting separately the dorsal and ventral pancreas to the duodenum. An exception to this pattern has been reported for two mammalian species, pig and ox, that do not have a connection between the ventral pancreas and the duodenum and so do not have an hepato-pancreatic common duct, an important duct found in all other mammalian species. Given that the hepato-pancreatic common duct is the site in all other mammals at which there are the largest numbers of niches for hepato-pancreatic stem/progenitors, it was necessary to re-evaluate the gross anatomy of the biliary tree and pancreatic duct system in pigs to confirm or to clarify these past reports.

**Fig. 1:**
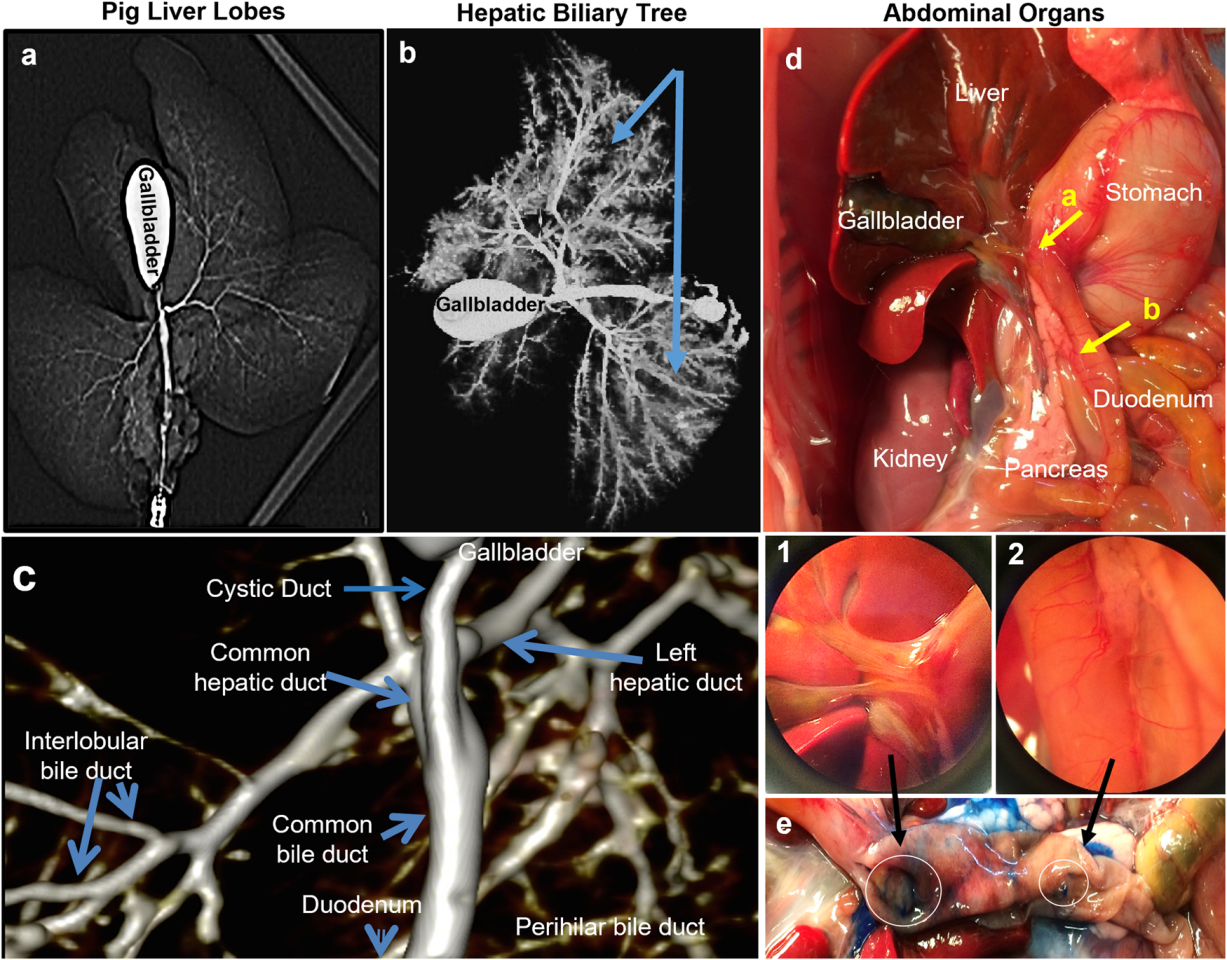
The anatomical features of the porcine biliary tree and its connections to liver, pancreas and duodenum. See also the Supplementary Videos 1–6 of the porcine hepatic and pancreatic biliary tree in the online supplement. **a**–**c** CT scans of porcine hepatic segments and biliary tree of a 7-week-old pig. **a** The 5 lobes can be distinguished from the image: 3 right lobes (right medial lobe, right lateral lobe, caudate lobe), and 2 left lobes (left lateral lobe, left medial lobe). Lipiodol ultra-fluid was pumped into the entrance of the common bile duct for the CT scan (red arrow) for ~1 h. **b** The schematic structure of porcine hepatic bile duct and gallbladder. Blue arrows are indicating the left division and right division of the liver. **c** A 3D reconstituted model of the porcine biliary tree was built according to the CT scan image by Osirix. The model shows the details of the biliary tree network in the pig’s liver and the connections with the gallbladder and duodenum. **d** Illustration of the abdominal organs for a 1-day-old piglet. The common duct and pancreatic duct enlargements in **d1** and **d2** have separate openings to the duodenum. India ink dye (blue) injections were made into the common bile duct for further confirmation. No blue ink was found outside of the site where the common duct enters the duodenum. This was demonstrated by injections at multiple sites into the pancreatic duct and pancreas parenchyma. **e** By splitting open the duodenum along the vertical axis, the minor duodenal papilla was located, indicated by blue ink, and is ~2 cm downstream to the hepatic bile duct connection to the primary duodenal papilla.

Newborn piglets euthanized for reasons unrelated to this study were collected for the purpose of mapping the gross anatomy of the intrahepatic, extrahepatic, and extra-pancreatic bile ducts. Computerized tomography (CT) scans were used to clarify their structures. The imaging reagent, Iopamidol 300, was pumped steadily into the common bile duct for imaging the structure of the entire porcine extrahepatic and intrahepatic biliary tree and their connections to the gallbladder and the cystic duct. (Fig. 1a, b). A reconstructed 3D image from the CT scan shown in Fig. 1c indicates the intrahepatic and extrahepatic biliary trees of pigs share a similar structure and gross anatomy to that in humans with the exception that in pigs there is no ventral pancreatic duct that joins with the common bile duct. Therefore, in pigs, the connections with the duodenum comprise that between the duodenum and the dorsal pancreas and separately that between the common bile duct from the liver and the duodenum.

To obtain greater clarity, the scans were repeated and combined with the use of India ink injections. Figure 1d presents the gross anatomy of the biliary tree of a newborn piglet. Shown is the common duct, formed by the merger of the cystic duct and the extrahepatic ducts (Fig. 1d1), as well as the dorsal pancreatic duct, that entered the muscle layers of the second segment of the duodenum (Fig. 1d2).

**Fig. 2:**
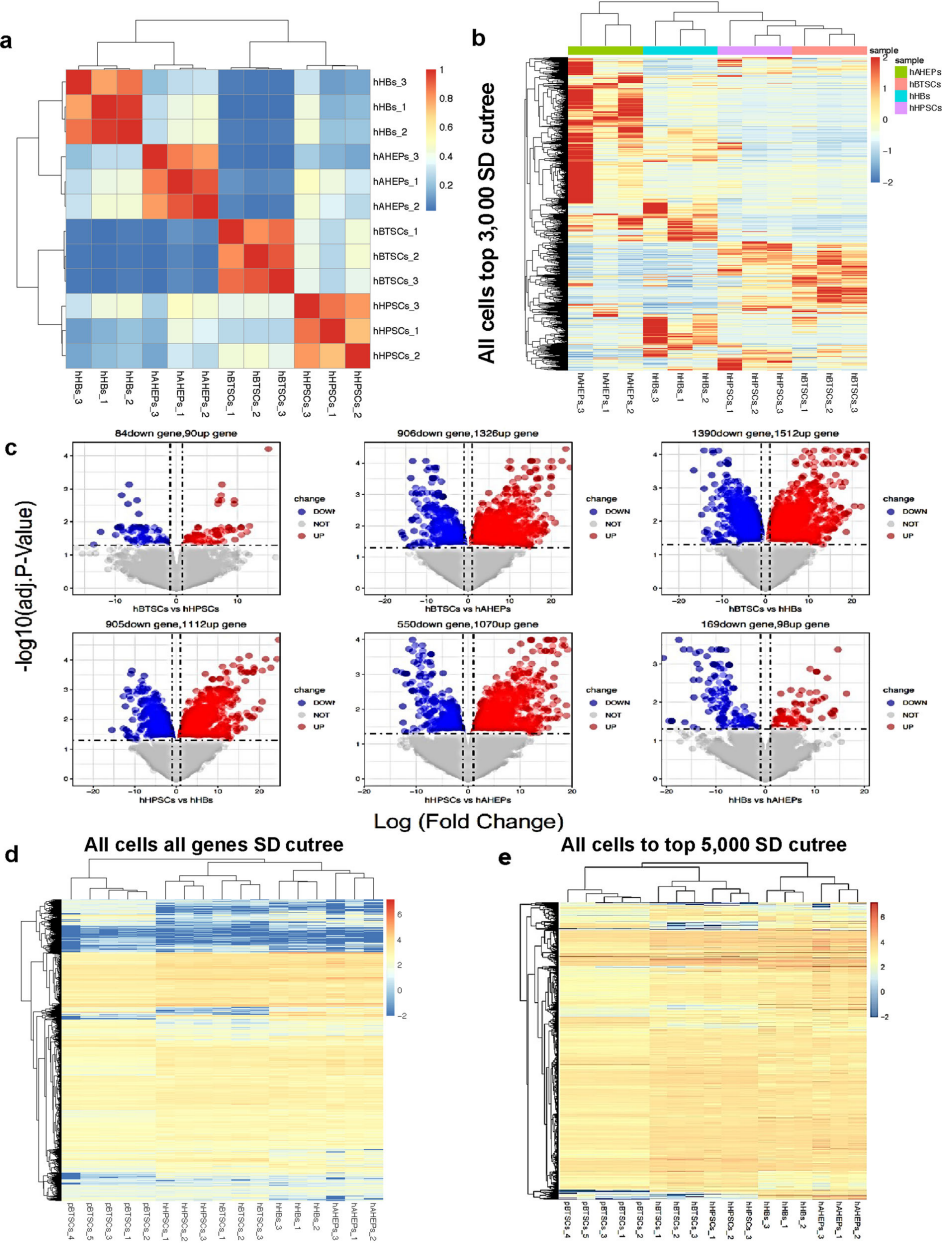
Genetic signature studies reveal similarities in porcine and human biliary tree stem cells. **a** Correlation analyses of hBTSCs, hHpSCs, hHBs and hAHeps presented as four distinct populations. **b** Expression and clustering analysis for the top 3000 genes of hBTSCs, hHpSCs, hHBs, and hAHeps. **c** Volcano Plots demonstrate the similarities and differences in gene expression between the cell types. The red spots indicate genes that are upregulated in the cell type, while the blue spots indicate the ones that are downregulated. The spots locate in further distance to the center of the graph are the genes that have the most distinct expression in one of the cell types. The adjusted *p* values are all < 0.05. Only the genes that had fold changes >2 (log_2_ fold change (log_2_FC) > 1) were colored in red or blue in the volcano plots. **d** The clustering analysis of the correlation heatmaps of all genes was the most distinct among all the five cell subpopulations. **e** The top 5000 genes that had the most significant standard deviation (SD) values were presented to show the similarities among pBTSCs and hBTSCs, hHpSCs versus hHBs and hAHeps.

We were less successful with the perfusion and imaging of the intrapancreatic duct system. The CT scans showed fragmented images of the ducts within the pancreas. The reasons for this are unknown. Furthermore, when injecting India ink into the common duct, no dye was observed in the pancreas and pancreatic duct, a finding that confirmed that there is no direct connection between the common duct and the ventral pancreatic duct (Fig. 1e). Serial sections of the adjacent site of the pancreas and common duct from 4 week-old piglets (Supplementary Fig. 1), provided further confirmation that there is no hepato-pancreatic common duct postnatally in pigs. A schematic summary of the anatomical features in pigs and a comparison to that in humans is given in Fig. 8.

See also the Supplementary videos 1–6 of the porcine hepatic, extrahepatic, and pancreatic biliary trees in the online supplement.

### Genetic signature studies comparing porcine and human stem/progenitors versus adult cells

**Fig. 3:**
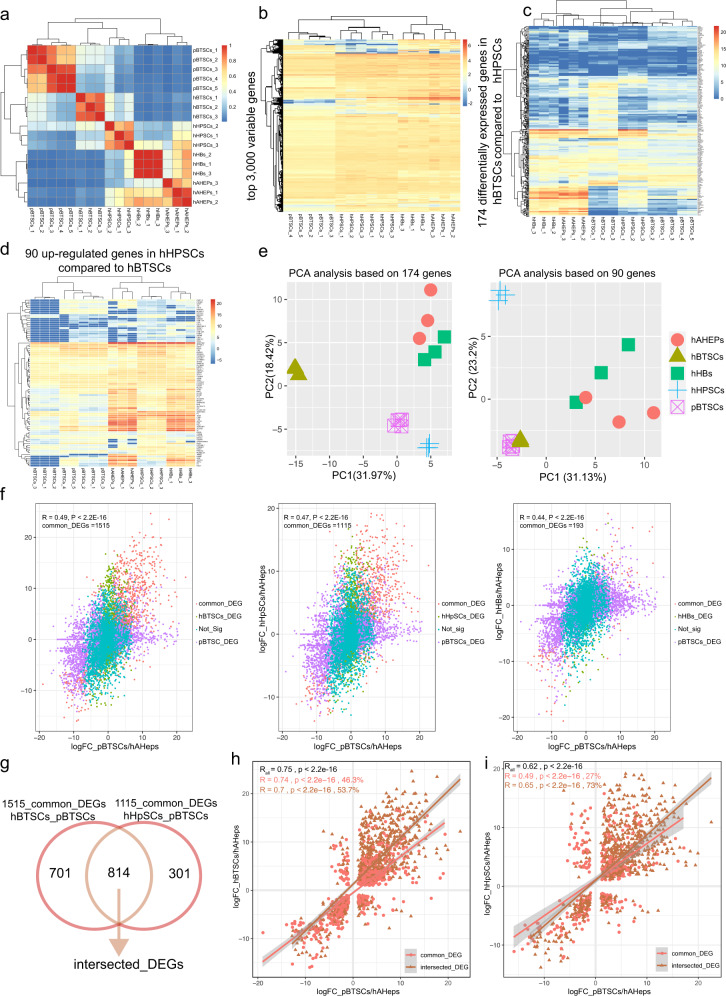
Genetic signature studies reveal similarities in porcine and human biliary tree stem cells. **a** Heatmap of the correlation of porcine biliary tree stem cells (pBTSCs, 5 samples) and four human cell subpopulations (hBTSCs, hHpSCs, hHBs, and hAHeps) were prepared for the similarity studies. The RNA-seq data of the five porcine biliary tree stem cells were mixed with the RNA-seq data of the four human cell subpopulations, and then the correlation analysis was done of all five types of cells for determining the lineage stages of the porcine cell subpopulations. The correlation heatmap of the five types of cells indicated two main clusters, pBTSCs cluster with hBTSCs and hHpSCs, while hHBs cluster with hAHeps. **b** Top variable 3000 genes among the five types of cells (pBTSCs, hBTSCs, hHpSCs, hHBs, and hAHeps) also show the same pattern as the correlation heatmap. pBTSCs share similar expression patterns with hBTSCs and hHpSCs, while a more distinguished pattern with precursor or mature lineages. **c** Clustering analysis based on 174 differentially expressed between hBTSCs and hHpSCs. **d** Clustering analysis of pBTSCs and four human lineages based on 90 up-regulated genes in hHpSCs compared to hBTSCs. **e** PCA analysis was processed for determining the equivalent lineage stage of pBTSCs in human hepato-biliary stem cell lineages. **f** pBTSCs, hBTSCs, hHpSCs, and hHBs were all compared with hAHeps. In all scatterplots, individual genes are plotted based on log_2_(fold change, FC) value. *R* is Pearson’s coefficient of significant genes. **g** Venn plot of common DEGs in Fig. 2f, showing common DEGs of hBTSCs, pBTSCs, and hHpSCs, and compared to hAHeps, respectively. The common DEGs among hBTSCs, pBTSCs, and hHpSCs were named as intersected DEGs. **h** and **i** Pearson’s coefficient of significant genes were used indicating further the similarities of DEGs (pBTSCs:hAHeps) compared to DEGs(hBTSCs:hAHeps) or DEGs(hHpSCs:hAHeps). *R* represents Pearson’s coefficient of DEGs*. R*_all_ = Pearson’s coefficient of all DEGs.

Genetic signature studies (Figs. 2 and 3) were used to guide us as to which markers could prove useful in identifying porcine stem/progenitors with findings to be compared with those found previously in the human biliary tree, liver, and pancreas^5,29,31,33^.

We prepared cell suspensions of the neonatal piglet tissues of the gallbladder, cystic duct, and common duct, and then from them isolated the porcine biliary tree stem cells (pBTSCs) for the analyses by RNAseq. Prior extensive characterizations of the human biliary tree, liver, gallbladder, duodenum, and pancreatic tissue included both immunohistochemistry and RNAseq^5,29,30,32,33,36,49,56^. The findings revealed distinctions in genetic expression between cells within the biliary tree versus the duodenum and stomach. The combination of the prior studies on human cells versus the current one on porcine cells enabled us to compare the findings in pigs with those from humans using the data from the cell fractions summarized below.

The surface expression of various antigens was used to identify particular subpopulations of cells isolated from the organs or tissues of pigs versus humans: those cells not expressing CD45—common leukocyte antigen (hemopoietic cells); EpCAM^+^—epithelial cell adhesion molecule; NCAM^+^—neural cell adhesion molecule on earlier stage cells; and ICAM-1^+^—intercellular adhesion molecule-1, found on later stage cells. The cell populations dominating the presumptive crypts had the highest expression of pluripotency genes and could be isolated and enriched by preparing cell suspensions from (a) fetal or neonatal tissue sources (liver, biliary tree, pancreas); (b) that were then immunoselected to be negative for CD45 expression; (c) with expression or lack of expression of EpCAM, and (d) with expression or lack of expression of either NCAM or ICAM-1. In summary:

CD45^−^, EpCAM^+^ cells from fetal or neonatal biliary trees (BTSCs)

CD45^−^, EpCAM^+^, NCAM^+^ cells from fetal or neonatal livers: hepatic stem cells (HpSCs)

CD45^−^, EpCAM^+^, ICAM-1^+^ cells from fetal or neonatal livers: hepatoblasts (HBs)

CD45^−^, EpCAM^−^ cells from fetal or neonatal livers and with cell sizes being >17/18 μm, are diploid hepatocytes (AHeps) [those prepared from pediatric or adult livers will include both diploid and polyploid hepatocytes, and the polyploid ones will be significantly larger in diameter].

The genetic traits in the subpopulations comparing human (h) subpopulations of [hBTSCs to hHpSCs to hHBs to hAHeps] were shown previously to be in patterns reflecting maturational lineage stages of cells from BTSCs to adult hepatocytes^52^ (Fig. 2a). The differential analysis showed that there are only 174 differentially expressed genes between hBTSCs and hHpSCs, and 267 genes between hHBs and hAHeps (Fold Change>2; adjusted *P*-value < 0.05) (Fig. 2b, c). Therefore, hBTSCs are more similar to hHpSCs, while hHBs resemble hAHeps based on correlation, hierarchical clustering, and differential analyses.

The species differences between humans (h) and pigs (p) were indicated in heat maps of all genes with the most significant SD values (Fig. 2d). In Fig. 3a when mixing the pBTSCs data set with that from the RNA-seq analyses of the four maturational lineage stages of human subpopulations [hBTSCs to hHpSCs, to hHBs, to hAHeps], the correlation analysis among the cell fractions showed that pBTSCs share more genetic similarities with hBTSCs and hHpSCs rather than with hHBs and hAHeps, confirming that the pBTSCs have a genetic signature appropriate for stem cells. Still, the heatmap of the top 5000 genes with the most significant SD values (Fig. 2e), and the top 3000 genes with the most significant SD values (Fig. 3b) indicated that pBTSCs’ genetics clustered with those of hBTSCs and hHpSCs and not with hHBs and hAHeps.

To confirm whether pBTSCs are more similar to hBTSCs or are nearer to hHpSCs, the 174 differentially expressed genes between hBTSCs and hHpSCs were obtained. The clustering analysis of pBTSCs with those 174 genes indicated pBTSCs are more similar to hHpSCs than to hBTSCs (Fig. 3c). Interestingly, when focusing only on the 90 up-regulated genes in hHpSCs, hypothesized to represent the stem cell characteristics of the hepatic lineages rather than that of the more primitive biliary tissue, pBTSCs clustered more with hBTSCs (Fig. 3d). Also when focusing on 84 genes highly expressed in hBTSCs, the pBTSCs are at quite an early stage so that these 84 up-regulated genes in hBTSCs did not offer distinctions with those in the other lineage stages studied (data not shown).

For the sake of visualizing the relationship primarily between pBTSCs, hBTSCs, and hHpSCs, the PCA analysis was also applied to the 174 differentially expressed genes and 90 up-regulated genes between hBTSCs and hHpSCs respectively. As is shown in Fig. 3e, pBTSCs were located between hBTSCs and hHpSCs alongside the Principal Component 1 (PC1), consistent with the aforementioned clustering results. Taken together, the genetic signature study indicated that pBTSCs are stem cells in an intermediate maturational stage between hBTSCs and hHpSCs.

### The pBTSCs are similar to hBTSCs

Given that pBTSCs were at an intermediate stage between hBTSCs and hHpSCs (Fig. 3a–e), we wanted to define further the maturational lineage stage of the pBTSCs. The differentially expressed genes (DEGs) in cells at the stages (pBTSCs, hBTSCs, hHpSCs, and hHBs) were compared to the genetic signature of adult parenchymal cells, hAHeps, to obtain their log_2_ Fold Change (log_2_FC), representing their genetic distance from hAHeps.

As shown in Fig. 3f, the significant correlation coefficients of whole genes are gradually reduced from 0.49, 0.47–0.44 when the log_2_FC of pBTSCs was compared to log_2_FC of hBTSCs, hHpSCs, and hHBs, indicating that pBTSCs are at a stage much closer to hBTSCs when compared to hAHeps. Of note, pBTSCs share 1515 common DEGs with hBTSCs, 1115 with hHpSCs, but only 193 with hHBs, which confirmed our finding that pBTSCs are at an intermediate stage between hBTSCs and hHpSCs (Fig. 3f). Interestingly when further comparing the 1515 common differentially expressed genes (DEGs) of pBTSCs and hBTSCs with the 1115 common DEGs of pBTSCs and hHpSCs, a total of 814 intersected genes were shared (Fig. 3g), implying that hBTSCs, pBTSCs, and hHpSCs were similar with each other, consistent with our previous study that hBTSCs are closer to hHpSCs while hHBs are closer to hAHeps (Fig. 3a–e).

Correlations of DEGs (pBTSCs: hAHeps) versus DEGs (hBTSCs: hAHeps) of the 814 intersected genes was *R* = 0.7 (*P* < 2.2E−16, Fig. 3h), while for DEGs (pBTSCs: hAHeps) versus DEGs (hHpSCs: hAHeps) was *R* = 0.65 (*P* < 2.2E−16, Fig. 3i). When including the other 701 genes of DEGs (pBTSCs: hAHeps) versus DEGs (hBTSCs: hAHeps) in Fig. 3g, a more significant correlation coefficient was obtained (*R*_all_ = 0.75, *P* < 2.2E−16, Fig. 3h). In contrast, when including the other 301 genes of common DEGs (pBTSCs: hAHeps) versus DEGs (hHpSCs: hAHeps), a lower correlation coefficient was achieved (*R*_all_ = 0.62, *P* < 2.2E−16, Fig. 3i), thereby revealing pBTSCs and hBTSCs have higher similarity than pBTSCs and hHpSCs no matter how analyzed by all common DEGs, intersected DEGs or the independent common DEGs. In summary, the correlation analyses indicated that pBTSCs are closer to hBTSCs rather than hHpSCs. Taken together, all the computational analyses above revealed that pBTSCs are genetically clustered most closely to hBTSCs.

### SOX2, SOX17, and PDX1 are highly expressed in hBTSCs and pBTSCs

**Fig. 4:**
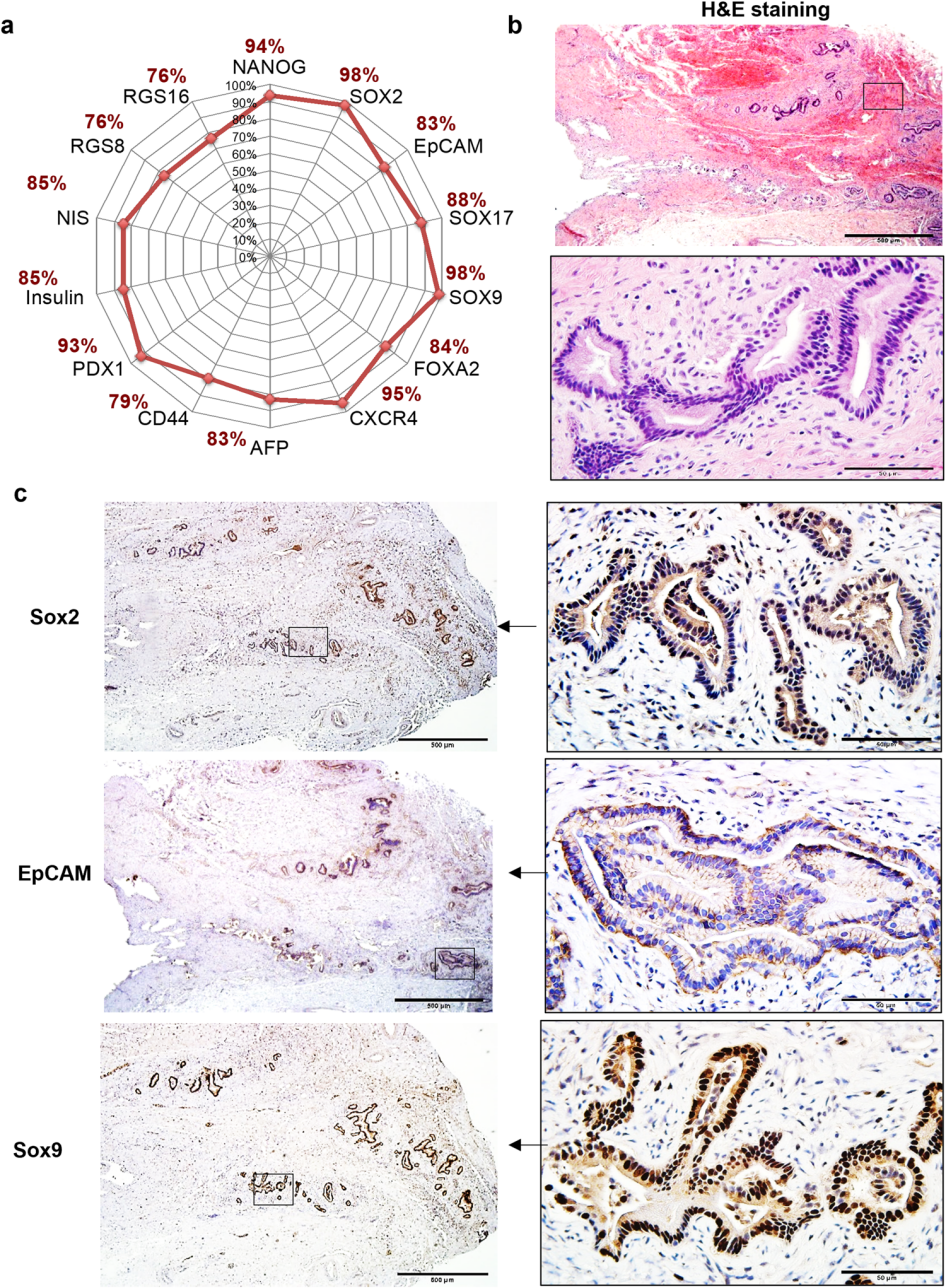
Comparison of porcine niches to those in the human biliary tree (see also Table 1). **a** A summary of the similarity of the stem cell and lineage cell markers between humans and pigs at both the gene level and protein level. **b** Paraffin sections of a 10% neutral-buffered-formalin-fixed and paraffin-embedded biliary tree tissue prepared from a 9-year-old, male human were used as positive controls for candidate antibodies screening and titration. H&E staining showed the preparation of the tissue preserved with the peribiliary glands (PBGs). **c** IHC staining for human biliary tree tissues of endodermal stem cell markers. IHC results with antibodies for porcine tissue study showed the PBGs expressed pluripotency markers (SOX2) and multiple endodermal stem cell markers (EpCAM and SOX9). Scale bars for low magnification images are 500 μm; for enlargement, images are 50 μm.

**Fig. 5:**
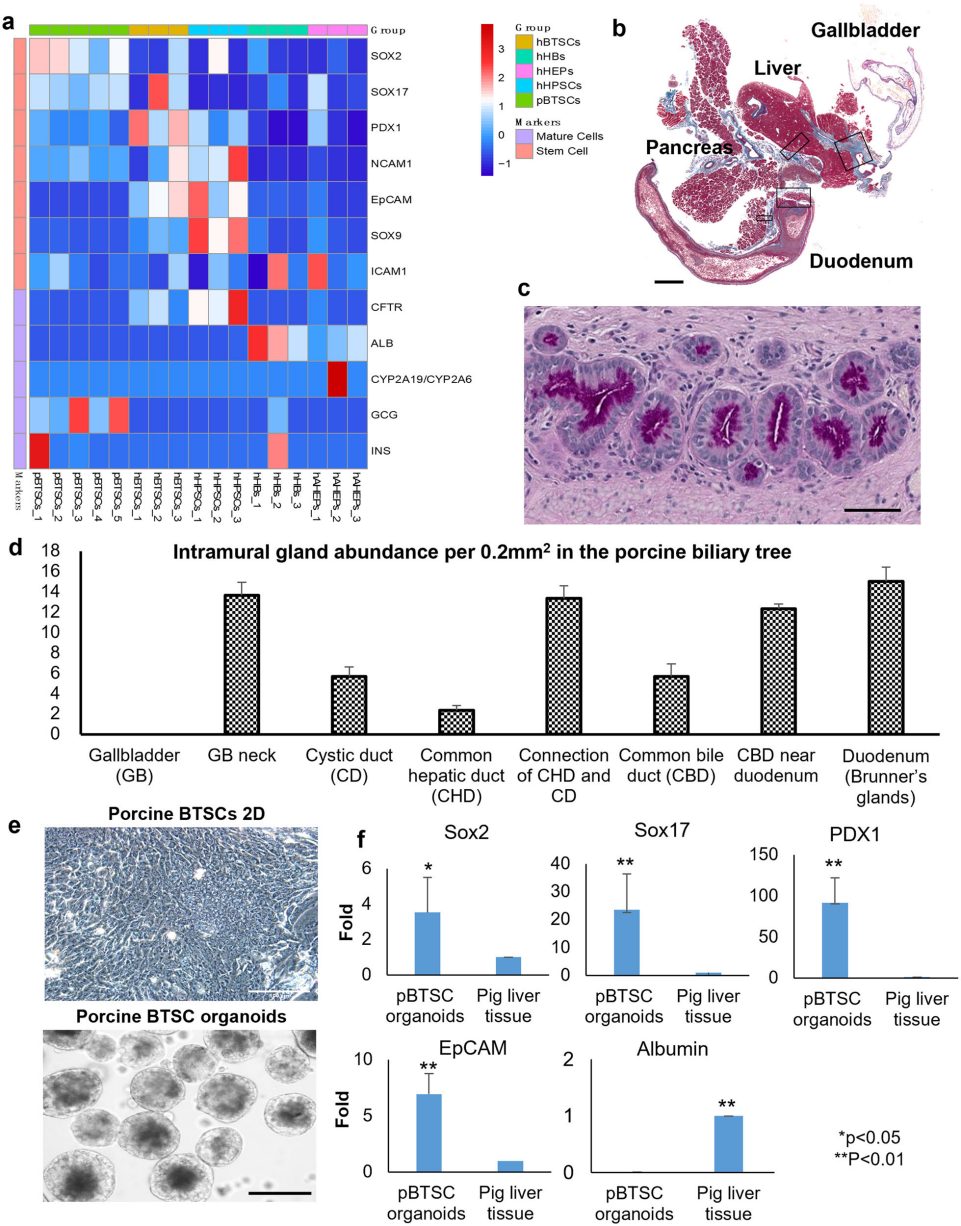
Selection of biomarkers to identify the porcine stem/progenitors. **a** Candidate biomarkers for the niches. Heatmaps of the expression pattern of stem cell markers versus maturation markers of porcine biliary tree stem cells (pBTSCs, five samples) and four human cell subpopulations (hBTSCs, hHpSCs, hHBs, and hAHeps) were used for selecting markers for the study of niches in porcine biliary trees. **b** Candidate locations for the niches. Sections of the large, formalin-fixed, paraffin-embedded (FFPE) blocks include the liver, gallbladder, pancreas, duodenum, and connective tissues from four 1-day-old piglets. They were studied for the distribution of PBGs among those organs and tissues. Scale bar = 3 mm. **c** PAS staining of a section of neonatal peribiliary glands (PBGs) at the duodenum papilla. Scale bar = 50 μm. **d** Quantitative analysis of the abundance of the niches at different sites in the biliary tree and duodenum. **e** Porcine biliary tree stem cells isolated from the biliary tree and placed in serum-free Kubota’s medium in 2D and 3D culture systems. The pBTSCs proliferate slowly in monolayer cultures; they form organoids within 12 h, and these can expand slowly. Scale bar = 100 μm. **f** The qPCR for endodermal stem cell markers versus maturation markers for pBTSCs. The cDNA of the pig liver tissue was used as a control to normalize the results. Results are presented as the mean ± standard deviation. The error bars indicate the standard deviation of triplicate values from three experimental replicates. *p < 0.05, **p < 0.01.

In Table 1 and Fig. 4a are summarized the lineage markers for the stem cell populations identified in human studies^5,30,39^. A comparison of gene sequences of the markers in pigs and humans was done. It was found that the genes have >80% similarities in the two species, making them logical candidates for defining the porcine hepato/pancreatic stem/progenitors (Fig. 4a). To confirm this hypothesis, we analyzed the expression patterns of those markers in human and porcine BTSCs versus in the fractions containing the more mature cells (Figs. 5a and 4). For both the human and porcine BTSCs, there is an expression of SRY-box transcription factor 2 (SOX2), SOX17, and pancreatic and duodenal homeobox 1 (PDX1) but no expression of mature parenchymal cell markers such as albumin (ALB), cystic fibrosis transmembrane conductance regulator (CFTR), or alpha-fetoprotein (AFP) (Fig. 5a).

**Table 1:** Lineage-stage specific markers for human biliary tree stem cells and hepato/pancreatic mature cells.

Human cell types	Lineage markers
Pluripotent stem cells	SOX2, NANOG, SALL4
Biliary tree stem cells	EpCAM, NIS, FOXA2, CD44, SOX17, PDX1, SOX9, CXCR4
Hepatocytes	HNF4A, AFP, ALBUMIN, TRANSFERRIN
Pancreatic islet and acinar cells	RGS8/16, INSULIN, TRYPSIN
Cholangiocytes	CFTR, AQUAPORIN-1, AQUAPORIN-9
Duodenal cell types	PDX1, CK8, CK9, MUC5

**Fig. 6:**
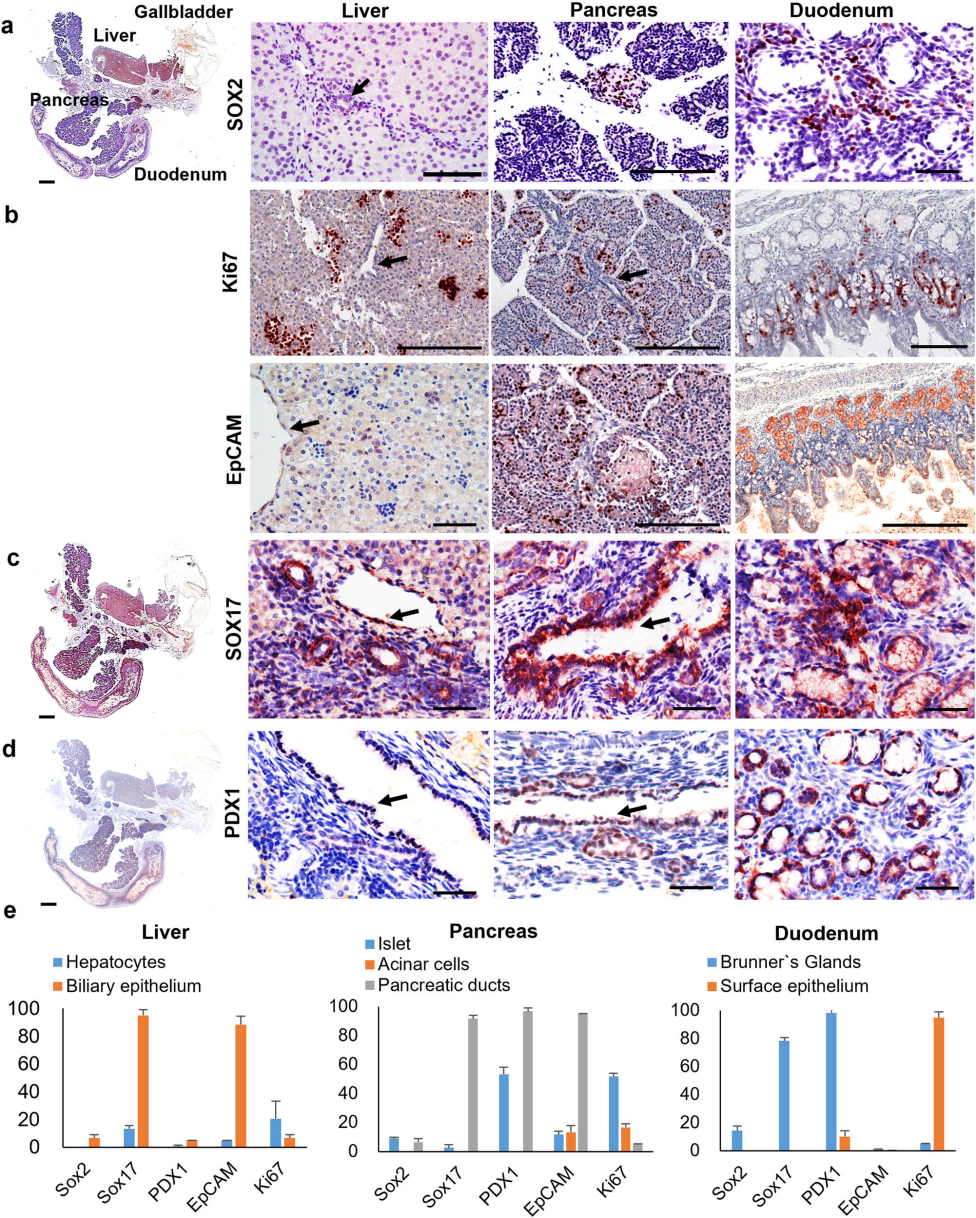
Stem cell niches in neonatal piglets. Localization in situ of stem cell niches in 1-day-old piglet livers, pancreas, and duodenum. Immunohistochemistry was processed on the sections of the Large-sized FFPE blocks of 1-day-old piglets using the set of stem cell markers (SOX2, SOX17, and PDX1) to identify the location of the stem cells niches in neonatal pig liver, pancreas, and duodenum. The left panel for every marker staining is the scaled image from the scanning of the entire section and is presented to give the landscape of the stem cell niches for endodermal stem cells. Scale bar for the enblock = 3 mm; for the enlargement images are 50 μm in all panels. **a** SOX2. Notice that the liver shows uniform dark staining in the section stained for SOX2. The SOX2^+^, SOX17^+^, PDX1^+^ stem cell niches can be observed primarily at the duodenum papilla where the hepatic common duct joins to the duodenum. For the pancreas, the zones are mostly in the glands in the pancreatic duct located at the pancreas head and partially in the next region of the pancreas, but not in the tail lobe. Cells doubly positive for Sox17 and PDX1 at the stem cell niches at the major papilla site at the duodenum. **b** Compared to the stem cell markers and EpCAM, Ki67 was found in the parenchyma of the liver, pancreas, and duodenum, and interestingly in distinct patterns in the three organs. **c** SOX17. Peribiliary glands at the large intrahepatic bile duct, the accessory pancreatic duct as well as the Brunner’s glands in the submucosa at the duodenum indicated SOX17^+^ signals. **d** PDX1. The large extrahepatic bile duct was found to be PDX1^−^, while the peribiliary glands in the accessory pancreatic duct, as well as the Brunner’s Glands in duodenum, were PDX1^+^. **e** Positive areas (percentages) of stem cell markers expressed by the hepatocytes and biliary epithelium in the liver; islet, pancreatic ducts, and acinar cells in the pancreas; Brunner’s Glands and surface epithelium in the duodenum. The error bars indicate the standard deviation of triplicate values from three experimental replicates.

**Fig. 7:**
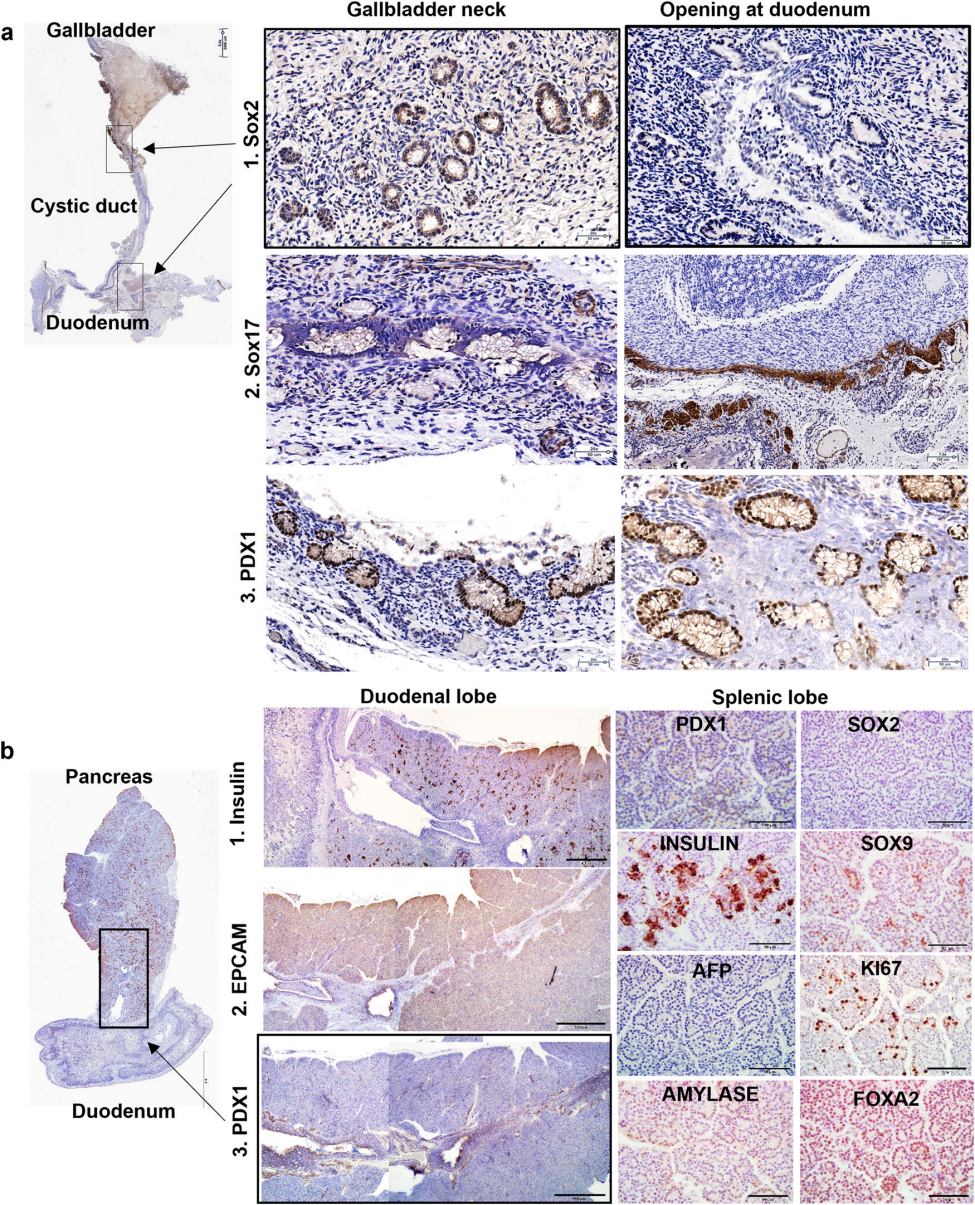
Niches in weanling (4-week-old) pigs. **a** Staining of SOX2, SOX17, and PDX1 showed a significant enrichment at the cystic duct and in the major papilla at the duodenum, illustrating the retention of stem cell niches for the adult pigs. Notice the positive signal for SOX17 in the deeper submucosa area of the duodenum. Scale bars for the panorama = 2.5 mm; for the enlargement images are 50 μm, except the Sox17 in the opening of the duodenum (130 μm). **b** Staining of the pancreas of the weanling piglet. The stem cell niches were observed at the pancreatic duct near the duodenum and in the duodenal lobe adjacent to the head of the pancreas, according to the staining of EpCAM and PDX1. They are not evident in the remaining lobes of the pancreas, where there are committed progenitors, but not stem cells, distinguished by expression of SOX9, FOXA2, INSULIN, but not PDX1, SOX2, and SOX17. Scale bars for the panorama = 4 mm; for all staining if the duodenal of the pancreas is 100 μm; for the splenic lobe of the pancreas are 50 μm.

Although there was a less clean separation in the pig fractions as compared with the human fractions, the results yielded confidence that, at the least, the genetic traits of pBTSCs were clearly defined, were very similar to hBTSCs, and so could be used to guide immunohistochemistry and in situ hybridization studies to identify the locations of the niches of BTSCs in surveys of the tissues of neonates (Fig. 6), weanling pigs (Fig. 7) and 7-week-old pigs (Supplementary Figs. 2 and 3).

### Stem/progenitor niches are identified with pBTSCs stem cell markers in piglets

A survey for the biomarkers identified for pBTSCs was done for the location of these cells in pigs of several ages and with special attention to where the largest numbers of them might be, given the absence of an hepato-pancreatic common duct^4,5,8,39^, a finding confirmed by our gross anatomy and initial microscopic studies. The survey was done of the entire biliary tree and pancreatic ducts plus the Brunner’s Glands in the duodenum. We prepared formalin-fixed liver, pancreas, biliary tree, gallbladder, and duodenum from piglets and embedded the organs and tissues from each neonatal piglet into a single, extra-large paraffin block, enabling us to evaluate all sites in a thin section of the relevant tissues (liver, pancreas, gallbladder, duodenum, and biliary tree) for biomarkers of stem cell traits. This significantly facilitated the survey to learn if the stem/progenitors exist and, if so, where they might be located.

We found patterns of niches in newborn piglets (Fig. 6), weanling (4-week-old) piglets (Figs. 7), and 7-week-old pigs (Supplementary Figs. 2 and 3). IHC staining for SOX2 (Fig. 6a), SOX17 (Fig. 6c), and PDX1 (Fig. 6d) was done on sections of the neonatal piglet tissue blocks (Fig. 6). Positive cells were found in the PBGs in the extrahepatic and intrahepatic biliary tree and in the portal zone of the liver but not in lobules within the liver, and none within the gallbladder. In the neonatal pig pancreas, they were found in the accessory duct and inside the pancreas in the PDGs of the large intrapancreatic ducts, those nearest to the duodenum.

We surveyed also the expression pattern for EpCAM and Ki67 to identify sites for proliferation versus quiescent cells in the organs using Ki67 to indicate proliferating cells and EpCAM^+^/Ki67^−^ cells to identify quiescent epithelial cells. Sequential sections of these zones were assessed. In Fig. 6b are shown the proliferating versus quiescent zones in the various tissues of neonatal piglets. In the liver, EpCAM^+^ cells were enriched in regions negative for Ki67, the ductal zone/portal zones of the livers. By contrast, Ki67^+^/EpCAM^−^ zones were found in the rest of the parenchymal and non-parenchymal zones of the neonatal pig liver.

For the neonatal pig pancreas, Ki67^+^ cells were found outside of the ductal zone and indicated developing acinar cells as part of exocrine development during the first week of postnatal life; the neonatal pig pancreatic islets were EpCAM^−^. EpCAM^+^ cells were found at the ductal zone and some of the acinar zones near the islets. For the neonatal piglets, the pancreas’s Islets and the ductal zones shared similar expression patterns for stem cell traits. However, the expression patterns showed a difference, which helped us to catalog the neonatal pancreas into islet portions, ductal portions, and the remaining portions that included the acinar cells and the connective tissue (Fig. 6b).

In the duodenum of neonatal piglets, Ki67^+^/EpCAM^−^ zones were found in the submucosa of the duodenum (near the intestinal crypts). By contrast, EpCAM^+^/Ki67^−^ zones were found in the mucosa. A summary of the findings is given in the table in Fig. 6e.

### The BTSC niches in 4-week-old pigs show an enrichment in the cystic duct

IHC staining for the stem cell biomarkers was done using weanling 4-week-old pigs. By this age of pigs, the size of the tissues precluded embedding all of the tissues into one block. In some of the images in Fig. 7, the extrahepatic biliary tree containing the gallbladder, cystic duct, and common bile duct was detached from the liver, while the pancreas, pancreatic duct, minor papilla, and duodenum were prepared separately.

In Fig. 7a pluripotency markers (e.g. SOX2) were used to survey the stem cell niches in the tissues (Fig. 7a1). Unlike that observed in newborn piglets, the gallbladder for the 4-week-old piglets presented as a much thicker, multi-layer structure in which the cystic duct, the neck of the gallbladder connecting to the common bile duct, contained PBGs with cells recognized by antibodies to SOX2. There were no other zones positive for these traits found in the rest of the biliary tree for the 4-week-old piglets. There were positive signals observed in the PBGs at the major papilla region of the duodenum but at levels lower than observed in the cystic duct.

The expression patterns of SOX17 (Fig. 7a2) and PDX1 (Fig. 7a3) were used also for locating the stem cells. Co-expression of PDX1 and SOX17 implicates co-precursors for the pancreatic and hepatic lineages. The results shown in Fig. 7a indicate that PDX1^+^ and SOX17^+^ signals were found in PBGs at the junction zone of the hepatic bile duct and cystic duct, as well as the cystic duct connection with the gallbladder, and at the major duodenal papilla region. Interestingly no other regions of the biliary tree indicated positive cells co-expressing PDX1 and SOX17.

To identify the stem cell niches in the pancreas, we used assays for various stem cell traits (e.g. EpCAM, PDX1, OCT4, SOX2, SOX9) as well as some mature markers (e.g. insulin and other islet hormones plus acinar cell markers such as amylase) (Fig. 7b). For piglets at 4-weeks of age, insulin expression was spread throughout the pancreas as clusters of cells but that did not yet have an adult islet morphology (Fig. 7b, 1). EpCAM and PDX1 positive signals were observed in the large pancreatic duct in the head of the pancreas near the duodenum (Fig. 7b, 2, 3). There were no cells positive for either EpCAM or PDX1 in the body or tail of the pancreas.

### Ex Vivo evidence of multipotency of pBTSC stem/progenitors

We isolated pBTSCs from porcine biliary trees and generated organoids by protocols established previously for human endodermal stem/progenitors^54^. The cells were maintained in serum-free Kubota’s Medium supplemented with 0.1% hyaluronans, conditions proven successful for ex vivo maintenance of organoids for weeks with the maintenance of stem/progenitor traits in hBTSCs, hHpSCs, and hHBs^54,55^. In Supplementary Fig. 4a, IHC staining of the organoids indicated that they were positive for biomarkers of stem/progenitors (SOX2, OCT4, SOX9, SOX17, PDX1, EpCAM) and were negative for biomarkers of more mature parenchymal cells (e.g. AFP, Insulin). Some cells on the surface (but not the interiors) of the organoids were weakly albumin positive. Subjecting the organoids to serum-free hepatic versus pancreatic differentiation conditions (Supplementary Table 3) resulted in cells expressing more mature hepatic or pancreatic markers depending on which conditions were used and, in parallel, there was the loss of the stem cell traits.

With defined conditions for hepatic fates, the cells converted to monolayers with classic hepatic morphology and exhibited increases in lipid depositions indicated with Oil Red O staining (Supplementary Fig. 4b). In data not shown, they also increased expression of albumin, cytochrome P450 2A19 (CYP2A19), and cystic fibrosis transmembrane conductance regulator (CFTR). With conditions for the pancreas, the organoids transitioned to aggregates or clusters with islet morphology and with an expression of Insulin and glucagon (Supplementary Fig. 4b, c). Detailed descriptions of the differentiation of the organoids under the hormonally defined medium and matrix conditions are given in other publications^54,55^.

### In vivo studies providing evidence of multipotency of porcine BTSC stem/progenitors

We recently confirmed the multipotent differentiation potential of pBTSCs by using patch grafting methods described previously to enable transplantation of organoids prepared from Green Fluorescent Protein^+^ (GFP^+^) transgenic pigs onto the livers versus pancreases of wild-type pigs or onto the livers or pancreases of mice with mutant liver or pancreatic conditions^54,55^. Shown in Supplementary Fig. 5 are representative data of the published study^54,55^. Patch grafting, a novel form of transplantation of organoids or of cell suspensions into solid organs, resulted in the engraftment of donor cells throughout much of the liver (or pancreas) within a week. It occurred in two phases: a remodeling phase in the first-week post-transplantation, followed in the second week by a differentiation phase of donor cells to mature cells^54^. In the remodeling phase, the liver’s Glisson’s capsule and the pancreatic capsule disappeared along with the histological structures for the organ caused by the production of multiple forms of matrix metalloproteinases (MMPs), especially MMP1 and MMP2, secreted by the donor cells, and enabling the organoids to engraft and migrate throughout the organ. Following rapid engraftment and migration, donor organoids showed evidence of maturation and differentiation of cells, a maturation that was paralleled with gradual muting of the expression of the MMPs, restoration of the organ capsules, and of the tissue-specific, matrix-dictated histological features along with rapid differentiation of donor cells to adult fates.

Patch grafts of GFP^+^ organoids onto the livers of wild-type pigs resulted in classic remodeling phenomena (Supplementary Fig. 5a) with loss of the Glisson’s capsule and histological structures and with donor GFP^+^ cells found throughout the liver within a week. At this time point, the donor cells were observed at early stages of differentiation towards a liver fate with an expression of HNF4α and α-fetoprotein (AFP) (Supplementary Fig. 4b). Within another week, the Glisson’s capsule was restored along with matrix component-dictated histological structures, and the donor cells had matured and expressed adult functions that included albumin, and not shown, also other markers of mature hepatocytes such as cystic fibrosis transmembrane conductance regulator (CFTR) and cytochrome P450 2A19 (CYP2A19).

Patch grafts of GFP^+^ organoids attached to the pancreases of wild-type pigs resulted in rapid engraftment of GFP^+^ donor cells (Supplementary Fig. 4a) that had migrated throughout much of the pancreas from the graft site^55^. Higher magnification images (Supplementary Fig. 4c) are of sections stained for insulin and with an antibody coupled to a red fluoroprobe; the section was also stained with 4′,6-diamidino-2-phenylindole (DAPI) for the nuclei. This enabled one to identify donor acinar cells (GFP^+^); host acinar cells (DAPI^+^); donor islets (yellow/orange color from the merger of GFP and red fluoroprobes); and host islets (red). The results show that the donor cells have given rise to a significant percentage of the mature acinar and islet cells in the host pancreas. The findings parallel those observed in extensive studies demonstrating the rescue of hosts from disease states such as tyrosinemia (liver) and type I diabetes (pancreas) by patch grafts of BTSC organoids^54,55^.

## Discussion

A network of co-hepato/pancreatic stem/progenitor niches is found in the intrahepatic, extrahepatic, and intrapancreatic biliary tree, and in Brunner’s Glands of the duodenum in pigs and humans^5,31,36^ with respect to genetic signatures, expressed phenotypic traits, and locations of their niches in situ. A distinction is that a large number of the stem/progenitors in humans is in the hepato-pancreatic common duct, but in pigs with no hepato-pancreatic common duct postnatally, they are found within pancreatic duct glands (PDGs) in regions of the dorsal and ventral pancreas near to the duodenum. Elsewhere in the porcine pancreas, and in the entirety of the human pancreas, only bipotent and unipotent committed progenitors were found.

In humans, there is evidence for life-long organogenesis of the liver and pancreas derived from the stem/progenitors in the biliary tree^5,30,31^. In pigs, the findings support that they exist postnatally for the developmental stages evaluated. Further studies are needed to learn if life-long evidence for stem/progenitors occurs also in pigs. Even with the findings to date, the findings qualify young pigs for translational, preclinical studies for stem cell therapies. Complementing the regenerative processes provided by the biliary tree-derived stem/progenitors are other mechanisms such as the plasticity of mature parenchyma and contributions by the terminal, diploid, Axin2^+^ hepatocytes^40,46^.

Markers for the co-hepato/pancreatic stem/progenitors in human tissues were well defined in prior studies^5,29,31–34,49,50^ but had not yet been characterized in pigs. Genetic signature studies enabled us to define these in human versus porcine tissues. Those selected included SOX2, implicating pluripotent cells, and SOX2^+^ cells co-expressing SOX9, implicating determined endodermal stem cells. Co-expression of SOX17 and PDX1 defined common ventral foregut progenitors^57^, cells giving rise to both the liver and pancreas. Therefore, a combination of SOX2, SOX9, SOX17, and PDX1 for IHC staining proved sufficient to locate the stem cell niches in the tissues.

PDX1 and SOX17 have known hepato/bilio/pancreatic stem cell markers that are detectable in murine embryos by e8.5. The expression of SOX17 in the ventral foregut endoderm region has been found essential for the segregation between liver and pancreato-biliary systems^58^. Deletion of SOX17 at e8.5 results in failure of expression of PDX1 and the loss of biliary structures and ectopic pancreatic tissue in the liver bud and common duct^59^. By contrast, overexpression of SOX17 suppresses pancreas development and promotes ectopic biliary-like tissue throughout the PDX1^+^ domain^60^.

In humans, there are cells co-expressing PDX1^+^ and SOX17^+^ in the PBGs of the cystic duct and in PBGs throughout the intrahepatic and extrahepatic biliary tree; the largest numbers being in the PBGs of the hepato-pancreatic common duct and in the PBGs of the large intrahepatic bile ducts^32,39^. In humans, in the gallbladder, that is devoid of PBGs, there are descendants of hBTSCs with muted stemness traits^30,32,33^. Also, in humans, there are descendants, hHpSCs, in canals of Hering, and their descendants, hHBs, next to canals of Hering, with late stemness traits and giving rise to committed hepatocytic and cholangiocytic progenitors that mature into hepatocytes and cholangiocytes^39–41^. The adult hepatic parenchymal cells undergo stepwise maturational changes in their phenotypic traits in parallel with changes in ploidy going from diploid cells near the portal triad to polyploid ones near the central vein^40,41^. The final stage in the liver, hepatocytes, bound on their lateral borders to the endothelia of the central vein, are diploid, and shown to be regulated by Wnt signaling, contributing to regenerative processes in the replacement of terminally differentiated, polyploid hepatocytes^46,47,61^.

An equivalent progression of stages in the pancreas has not yet been done, though progression at least from the BTSCs to bipotent pancreatic ductal progenitors in pancreatic duct glands has been established and with those near the duodenum being found to be earlier stages versus those elsewhere in the pancreas having traits suggestive of later maturational stages^30,32,36,49^.

Brunner’s Glands, submucosal duodenal glands, are composed of neutral, mucin-secreting, cuboidal to columnar cells with basally located nuclei and are comprised of cells arranged in lobules containing thin fibrous septa^16,36^. To date, much is known about the ways in which Brunner’s glands participate in ion transport and production of secreted factors, such as epidermal growth factor receptor ligands^15,16^. However, until recently, nothing was known of them as a starting point for the network of stem/progenitors in hepatic and pancreatic biliary tree branches. Recent studies of these duodenal glands in humans indicate that they contribute to pancreatic and hepatic organogenesis^34,36^. The studies here indicate that such stem/progenitors in the porcine duodenal glands exist also, but further investigations are needed to define whether further parallels exist with the findings in Brunner’s Glands’ duodenal stem cells in humans^36^.

In pigs, four major sites of stem cell niches were identified with expression of SOX2, SOX9, SOX17, and PDX1. Primary sites were at the connection from the duodenum to the common bile duct and to the pancreatic accessory duct for all ages of pigs studied. A third site with large numbers of stem/progenitors is in the cystic ducts in older animals (>4 weeks of age). The fourth site, unique to pigs, is in the PDGs, in regions of the dorsal and ventral pancreas near to the duodenum. Elsewhere in the porcine pancreas, the precursors identified are committed unipotent and bipotent progenitors. By contrast, in humans, with rare exceptions, there are no stem cells within the human pancreas proper at all^31,49,50,62^. Rather, the cells found in humans have a negligible expression of pluripotency genes and comprise only bipotent or unipotent progenitors^49,50,62,63^.

An unsolved mystery is the missing hepato-pancreatic common duct in pigs^64^. During porcine embryo development, the rudiments of the nascent liver and pancreas develop similarly to that in all other mammals with there being a duct between the duodenum and the liver and two between the duodenum and the pancreas, one for the dorsal pancreas and one for the ventral pancreas. Differences in pigs develop at the 120 cm stage of embryos, following which the ventral pancreatic duct disappears, and the hepatic common duct and dorsal pancreatic duct connect separately with the duodenum. This is demonstrated in the animation in the online supplement comparing the development of the connections in pigs versus most mammals (represented in the animation by the findings in dogs in Supplement Video 5).

We further confirmed this finding by evaluating pigs at several developmental ages. Neonatal piglets proved an excellent first anatomical model that was easier to trace the connections of the liver and pancreas to the duodenum due to the scarcity of fat tissue around the abdominal organs as compared to that found in adult animals. Both the anatomical and histological results showed no evidence of the existence of the ampulla for the hepatopancreatic common duct to the ventral pancreas after the 120 cm stage in fetuses and in neonates, confirming that from prior studies^1,3,64^. We learned that hepato/pancreatic co-stem/progenitors do exist in pigs, with many of the locations being the same as in humans, but with distinct subpopulations being in PDGs within the pancreas and near the duodenum.

Another mystery that remains is how acinar cell-derived factors, the digestive enzymes, gain access to the gut. The only certain connection in pigs is the accessory pancreatic duct from the dorsal pancreas to the duodenum. We are incredulous that all the digestive enzymes from the porcine pancreas are delivered through that channel, especially given the extraordinary size of the pancreas in adult pigs. Although considerable efforts were spent to identify any remnants postnatally of the ventral pancreatic duct in serial sections of the pancreas and of the duodenum and even in regions of the small intestine, it was never found (Supplementary Fig. 1). We speculate that especially the close apposition of the duodenum to the head of the dorsal and ventral pancreas and to the initial regions of the small intestine might suggest an alternative, novel connection(s) allowing passage of pancreatic factors into the duodenum or intestine, a hypothesis to be addressed in future studies and possibly requiring electron microscopy to identify such passages.

**Fig. 8:**
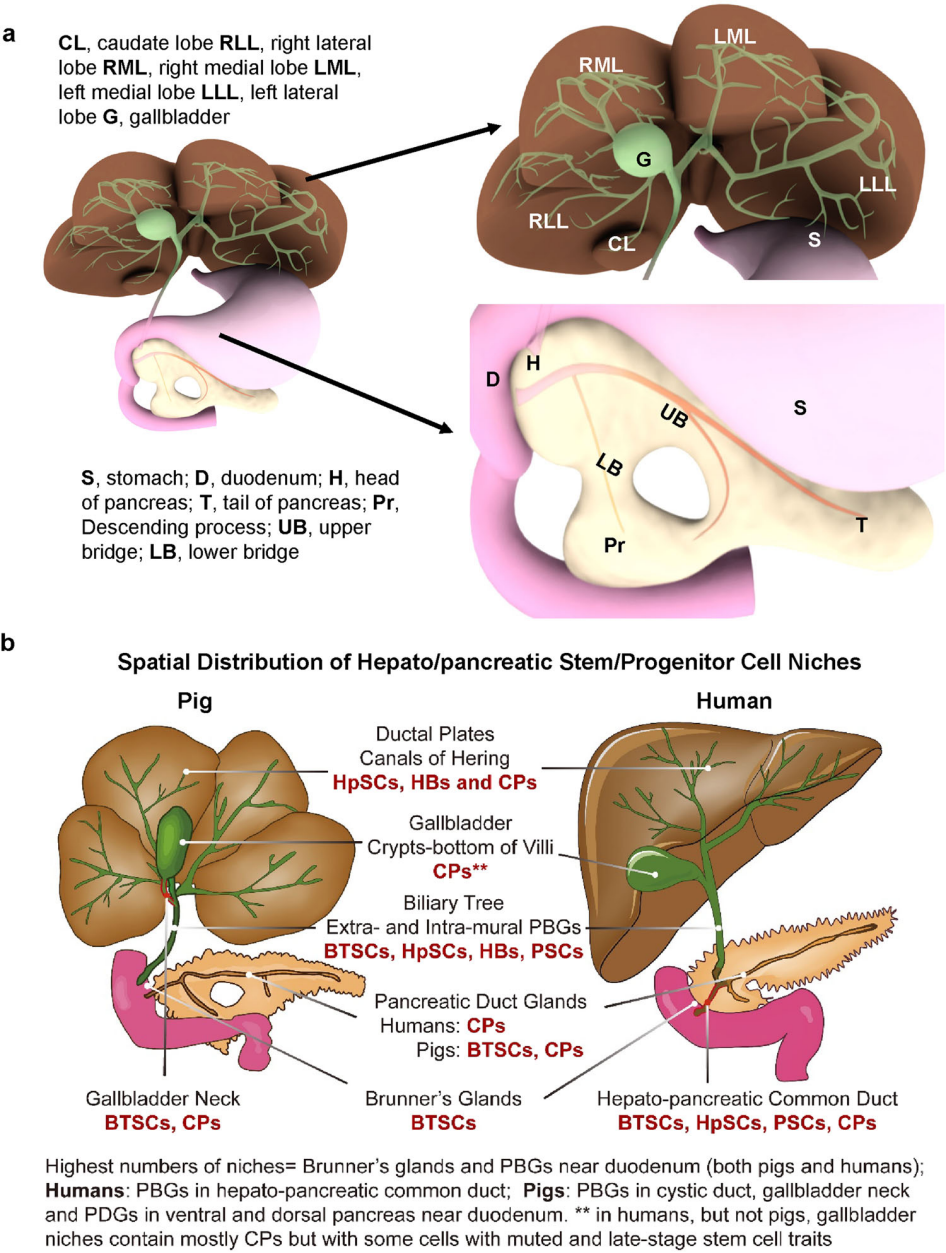
Summary of the distinctions in anatomy and in the location of the stem/progenitor niches in the human versus porcine biliary trees. **a** Illustration of the lobule patterns for porcine liver, biliary tree, and pancreas. Abbreviation for enlarged liver lobes: CL caudate lobe, RLL right lateral lobe, RML right medial lobe, LML left medial lobe, LLL left lateral lobe, G gallbladder; Abbreviation for enlarged pancreas: S stomach, D duodenum, H head of pancreas, T tail of pancreas, Pr Descending process; UB upper bridge, LB lower bridge. **b** Locations of the stem/progenitor cell niches. The highest numbers of the hepato/pancreatic co-stem/progenitor niches are in the submucosal duodenal glands (also called Brunner’s Glands), in peribiliary glands (PBGs) throughout the biliary tree and in the accessory duct to the dorsal pancreas; the ones in PBGs near the liver transition to hepatic stem cell niches in the canals of Hering connecting into the parenchyma in the liver acinar plates. The major distinctions between pigs and humans are that humans, but not pigs, have the hepato/pancreatic common duct connecting the duodenum to the ventral pancreas. It is the location of the highest number of stem/progenitor cell niches in the biliary tree in all mammals, other than pigs or ox. Pigs, but not humans, have stem cell niches in the pancreatic duct glands (PDGs) within the pancreas proper, those PDGs that are in regions near the duodenum. Both humans and pigs have committed bipotent and unipotent progenitors, but not the stem cells, in PDGs elsewhere in the pancreas. There are also progenitors in the gallbladder, though the ones in the human gallbladder retain some traits of stemness albeit muted relative to the traits in the cells at the base of the PBGs (near the fibromuscular layer), the presumptive stem cell niches in the biliary tree. Further details are provided in Supplementary Figure 6.

In summary, despite the primary distinction of pigs (and the ox) with respect to all other mammals in not having an hepato-pancreatic common duct, pigs are like all mammals studied and have a network of co-hepato/pancreatic stem/progenitors capable of maturing into liver or pancreas, a network comparable to the one in humans. Pigs are commonly used in large animal models for translational/preclinical studies due to the similarity of organ size and metabolic patterns between humans and pigs. Most aspects of the networks of stem/progenitors in humans and pigs are remarkably similar in location (summarized in Fig. 8) and phenotypic traits, in the methods successful for their isolation, cryopreservation, and culture, and in the strategies for eliciting their differentiation to adult fates ex vivo and in vivo. Species-specific distinctions include:

1. There are larger numbers of PBGs throughout the biliary tree in humans.
2. All the PDGs in humans contain only committed unipotent and bipotent progenitors.
3. Pigs have stem cells both in PBGs and in a subset of the PDGs, those near the duodenum. Elsewhere in the porcine pancreas, the PDGs contain only committed unipotent and bipotent progenitors.
4. In older pigs, there are PBGs in the cystic duct.

The pig model used has a highly heterogeneous genetic background and parallels the heterogeneous genetics in human populations. Considering that there are more than 40 breeds of domestic pigs, there might be distinct patterns of stem/progenitors in the biliary tree and duodenum for the other breeds, a suggestion worthy of future studies. The studies to date support the conclusion that pigs should be ideal models for translational/preclinical studies relevant to the cell therapy potential of the stem/progenitors in normal and diseased conditions. We hope to do such studies focused on patch grafting of organoids of BTSCs for the treatment of hepatic and pancreatic diseases. In related studies, we found that patch grafting proved efficient in the transplantation of organoids into the liver and pancreas of pigs and in murine experimental models and were able to correct disease states such as tyrosinemia (liver) and type I diabetes (pancreas)^54,55^. Thus, we anticipate that pigs should be ideal hosts for preclinical studies for cell therapies for the liver and pancreas as representative endodermal organs.

## Methods

Companies providing equipment, reagents and/or supplies: Abcam, Cambridge, MA; ACD Labs, Toronto, CA; Acris Antibodies, Inc), San Diego, CA; Advanced Bioscience Resources, Inc (ABR), Rockville, MD; Agilent Technologies, Santa Clara, CA; Alpco Diagnostics, Salem, NH; Applied Biosystems, Foster City, CA; BD Pharmingen, San Jose, CA; Becton Dickinson, Franklin Lakes, NJ; Bethyl Laboratories, Montgomery, TX; BioAssay Systems, Hayward, CA; Cambridge Isotope Laboratories, Tewksbury, MA; Biotime, Inc, Alameda, CA; Carl Zeiss Microscopy, Thornwood, NY; Carolina Liquid Chemistries, Corp., Winston-Salem, NC; Charles River Laboratories International, Inc, Wilmington, MA; Chenomx, Inc, Alberta, Canada; Cole-Parmer, Court Vernon Hills, IL; DiaPharma, West Chester Township, OH; Fisher Scientific, Pittsburgh, PA; Gatan, Inc, Pleasanton, CA; Illumina, San Diego, CA; Ingenuity, Redwood City, CA; Life Technologies Corp., Grand Island, NY; Leica, Washington, DC; LifeSpan Biosciences, Inc, Seattle, A; Molecular Devices, Sunnyvale, CA; Olympus Scientific Solutions Americas Corp., Waltham, MA; PhoenixSongs Biologicals (PSB), Branford, CT; Polysciences, Inc, Warrington, PA; Qiagen, Germantown, MD; R&D Systems, Minneapolis, MN; RayBiotech, Norcross, GA; Roche Diagnostics, Mannheim, Germany;Santa Cruz Biotechnology, Inc), Dallas, TX; Sigma-Aldrich, St. Louis, MO; Sofregen, Medford, MA; Takara, Otsu, Japan; Tousimis Research Corp., Rockville, MD; Triangle Research Labs (TRL), Research Triangle Park, NC; Umetrics, Umea, Sweden; Varian Medical Systems, Inc), Palo Alto, CA; Vector Laboratories, Burlingame, CA; VWR Scientific, Radnor, PA.

Ethical statements summarize how the animals were maintained. All animal experiments were performed in strict accordance with the approved Institutional Animal Care and Use Committees of North Carolina State University (NCSU) in Raleigh and at the University of North Carolina (UNC) in Chapel Hill and so have followed the principles outlined in the Declaration of Helsinki for all human or animal experimental investigations. All procedures used in animal studies and those involving the use of human tissues were approved by The Institutional Animal Care and Use Committee (IACUC) and Institutional Review Board (IRB) committees at both institutions: the UNC and at the College of Veterinary Medicine at NCSU. The IACUC approval numbers are 16-316.0 and 17-225.0. Studies for human cells in this project were evaluated by the IRB committee and were provided an IRB approval number of 97-1063.

Human samples include information on each sample plus information general for all the samples and provided in detail in prior studies:^39,41,42^

Fetal liver tissue was provided by an accredited agency (Advanced Biological Resources, San Francisco, CA) from fetuses between 18 and 22 weeks gestational age obtained by elective terminations of pregnancy. The research protocol was reviewed and approved by the IRB for Human Research Studies at UNC. All samples were screened for various pathogens and only those free of these were accepted for the research studies.

Postnatal livers were obtained from cadaveric neonatal, pediatric, and adult donors and were obtained through organ donation programs via UNOS. Those used for these studies were considered normal with no evidence of disease processes including following screening for pathogens. Informed consent was obtained from next of kin for the use of the livers for research purposes, protocols received IRB approval, and processing was compliant with Good Manufacturing Practice.

Pigs samples were from animals maintained at the facilities at the College of Veterinary Medicine at North Carolina State University (NCSU, Raleigh, NC). Some of them were used as hosts or as donors for cells. Surgeries, necropsies, and the collection of all biological fluids and tissues were performed at these facilities.

Porcine hosts used for the grafts were a mixture of six different breeds: a six-way cross consisting of Yorkshires, Large Whites, Landraces (from the sows), Durocs, Spots, and Pietrans (from the boars). This highly heterogeneous genetic background is desirable in that it parallels the heterogeneous genetic constitutions of human populations^65^. The host animals were all females, ~6 weeks of age and ~15 kg.

Green fluorescent protein (GFP)^+^ transgenic pig donors were established carrying an H2B histone-eGFP transgene. The GFP^+^ donor animals were obtained by breeding a transgenic H2B-GFP boar with a wild-type gilt by standard artificial insemination^66^. The model was developed via CRISPR-Cas9-mediated homology-directed repair (HDR) of IRES-pH2B-eGFP into the endogenous β-actin (ACTB) locus. The transgenic animals show ubiquitous expression of pH2B-eGFP in all tissues. Fusion of the GFP to H2B results in the localization of the GFP marker to the nucleosome and allows clear nuclear visualization as well as the study of chromosome dynamics. The founder line has been analyzed extensively and ubiquitously, and nuclear-localized expression has been confirmed. In addition, breeding has demonstrated transmission of the H2B-GFP to the next generation. All animals were healthy, and multiple pregnancies have been established with progeny showing the expected Mendelian ratio for the transmission of the pH2B-eGFP. The male offspring were genotyped at birth, and those that were positive for the transgene were humanely euthanized for tissue collection and isolation of donor cells.

Genotyping of porcine animals was done. For each donor and recipient animal, the swine leukocyte antigen class I (SLA-I) and class II (SLA-II) loci have been polymerase chain reaction (PCR) amplified using primers designed to amplify known alleles in these regions based on the PCR-sequence-specific-primer strategy^18^. The system consists of 47 discriminatory SLA-I primer sets amplifying the SLA-1, SLA-2, and SLA-3 loci, and 47 discriminatory SLA-II primer sets amplifying the DRB1, DQB1, and DQA loci^67^. These primer sets have been developed to differentiate alleles by groups that share similar sequence motifs and have been shown easily and unambiguously to detect known SLA-I and SLA-II alleles. When used together, these primer sets effectively provided a haplotype for each animal that was tested, thus providing an assay to confirm easily a matched or mismatched haplotype in donor and recipient animals.

Surgeries on the pigs were done using anesthesia induced by administering a combination of ketamine/xylazine (2–3 mg/kg weight each) injected IV or 20 mg/kg ketamine plus 2 g/kg xylazine IM, and were maintained by isoflurane in oxygen administered via a closed-circuit gas anesthetic unit. In the grafts on the liver and general surgical procedures, the pigs were positioned in dorsal recumbency, and the ventral abdomen was clipped from xyphoid to pubis. The skin was aseptically prepared with alternating iodinated scrub and alcohol solutions. After entry into the surgery suite, preparation of the skin was repeated using a sterile technique, and the area was covered with a topical iodine solution before the application of sterile surgical drapes. The surgeons used an appropriate aseptic technique. A mid-ventral incision was made through the skin, through subcutaneous tissues and *linea alba*, starting at the xiphoid process and extending caudally 8–12 cm. The left hepatic division was exposed, and a 3 × 4.5 cm patch graft was applied to the ventral surface of the liver and containing a 1X hyaluronan hydrogel (~60 Pa) with embedded BTSC organoids and placed onto the backing containing 10X hyaluronan hydrogel (~760 Pa); the patch was placed in direct contact onto the surface of the liver capsule. The patch graft was sutured to the liver using 4–6 simple, interrupted sutures of 4–0 polypropylene. The exposed surface of the graft was then treated with 2 ml of 2X hyaluronan hydrogel (~200–300 Pa), a level of rigidity that was fluid enough to permit it to be painted or coated onto the outside of the graft; it served further to minimize adhesions from neighboring tissues. Following placement of the surgical graft, the *linea alba* was closed with a simple continuous suture using 0-PDS. The *linea* was blocked with 2 mg/kg 0.5% bupivacaine, IM. The subcutaneous tissues and skin were closed with continuous 2–0 PDS and 3–0 Monocryl sutures, respectively. Tissue adhesive was placed on the skin surface.

For grafts on the pancreas, surgical procedures for pigs made use of a graft that can be any size that accommodates the dimensions of the pancreas. For exposure of the pancreas, stay sutures were placed in the descending duodenum. The graft was placed onto the head of the pancreas and adjacent to the duodenum with the soft hyaluronan (~60 Pa) hydrogel containing the organoids in direct contact with the pancreas, and with the more rigid hyaluronan hydrogel (~760 Pa) within the silk backing. We used sutures at the four corners (4–0 Prolene); the first two corner sutures were placed in the descending duodenum; the other 2 were placed in the mesentery and avoiding the pancreatic parenchyma (to minimize any propensity for autolysis by the pancreas). The serosal surface of the graft was then covered with 2X hyaluronan (~200–300 Pa) hydrogel to minimize adhesions. The abdomen was closed with continuous absorbable (PDS) sutures in the linea, subcutaneous and subcuticular layers. Tissue adhesive was used on the skin to avoid skin sutures.

Immunosuppression was required since the transplants from the transgenic pigs to the wild-type recipients were allogeneic. The immune-suppression protocols used were ones established by others^68,69^. All pigs received oral dosages of the immunosuppressive drugs, Tacrolimus (0.5 mg/kg) and Mycophenolate (500 mg) twice daily, beginning 24 h prior to surgery. The drugs were given continuously for the entire experimental period. These could be given to the animals easily if mixed with their favorite foods.

Isolation of normal tissues was done from newborn piglets and from older, 12-week-old pigs. Newborn piglets weighed ~10 lbs; the older pigs, 12 weeks of age, were ~50–60 lbs. Pigs were sacrificed by penetrating captive bolt euthanasia followed by jugular exsanguination. The method meets the recommended guidelines of the American Veterinary Medical Association for euthanasia in pigs^65^. A listing of donors used for biliary tissues from piglets or adult pigs is given in Supplementary Table 4.

All media were sterile filtered (0.22 µm filter) and kept in the dark at 4 °C before use. Basal medium and fetal bovine serum (FBS) were purchased from GIBCO/Invitrogen. All growth factors were purchased from R&D Systems. All other reagents, except those noted, were obtained from Sigma.

Cell wash buffers consisted of 500 mls of basal medium (e.g. RPMI 1640; Gibco # 11875-093) were supplemented with 0.5 g of serum albumin (Sigma, # A8896-5G, fatty-acid-free), 10^−9^ M selenium, and 5 mls of antibiotics (Gibco #35240-062, AAS). It was used for washing tissues and cells during processing.

Collagenase buffer consisted of 100 mls of cell wash supplemented with collagenase (Sigma # C5138) with a final concentration of 600 U/ml (R1451 25 mg) for biliary tree (ducts) tissue and 300 U/ml (12.5 mg) for organs (e.g. liver).

Kubota’s Medium, a wholly defined, serum-free medium designed originally for hepatoblasts^70^, and then found successful for the maintenance of biliary tree stem cells, hepatic stem cells, and pancreatic stem cells and progenitors (and later found successful in general for all endodermal stem/progenitors assessed), was used to prepare cell suspensions, organoids, and hyaluronan hydrogels. This medium consists of any basal medium (here being RPMI 1640) with no copper, low calcium (0.3 mM), 1 nM selenium, 0.1% serum albumin (purified, fatty-acid-free; fraction V), 4.5 mM nicotinamide, 0.1 nM zinc sulfate heptahydrate, 5 µg/ml transferrin/Fe, 5 µg/ml insulin, 10 µg/ml high-density lipoprotein, and a mixture of purified free fatty acids that are presented complexed with fatty-acid-free, highly purified albumin. The free fatty acid mixture was comprised of palmitic acid, palmitoleic acid, stearic acid, oleic acid, linoleic acid, and linolenic acid. Kubota’s medium is available commercially from *PhoenixSongs Biologicals* (Branford, CT).

Hyaluronans (HAs) used included the soluble, long-chain forms of HA (Sigma Catalog #52747) and were used in the stabilization of organoid cultures and in cryopreservation of cell suspensions^71,72^. Those used to make the hydrogels, thiol-modified HAs, were obtained from Glycosan Biosciences, formerly a subsidiary of Lineage Cell Therapeutics (Alameda, CA), and are now offered in non-clinical grade form from Advanced Biomatrix (Carlsbad, CA) and in the clinical grade form from Sentrex Animal Care (Salt Lake City, UT) (G. Prestwich, personal communications). The components for these thiol-modified HAs were made by a proprietary bacterial-fermentation process using *Bacillus subtilis* as the host in an ISO 9001:2000 process (www.biopolymer.novozymes.com/). The components were produced by Novozymes under the trade name, HyaCare®, and are 100% free of animal-derived materials and residual organic solvent. No animal-derived ingredients are used in the production; there are very low protein levels and no endotoxins. The production follows the standards set by the European Pharmacopoeia. The HA hydrogels were prepared using Glycosil (HyStem® HAs, ESI BIO-CG313), the thiol-modified HAs that can be triggered to form disulfide bridges in the presence of oxygen, or by forming thio-ether linkages using polyethylene glycol diacrylate (PEGDA). Glycosil® is reconstituted as a 1% solution of thiolated HA in 1% phosphate-buffered saline (PBS) using degassed water, or, in our case, in serum-free Kubota’s Medium. Upon reconstitution, it remains liquid for several hours but can undergo some gelation if exposed to oxygen. More precise gelation occurs with no temperature or pH changes if Glycosil is treated with a cross-linker such as PEGDA causing gelation to occur within a few minutes^73–76^.

The level of cross-linking is the primary contributor to the level of stiffness (viscoelasticity) or rigidity and can be controlled by adjusting the ratio of the thiol-modified hyaluronans to PEGDA^76^. In prior studies, hepatic stem cell populations were tested in HA hydrogels of varying levels of rigidity and were found to remain as stem cells, both antigenically and functionally (e.g. with respect to the ability to migrate and to the expression of stem cell-associated genes such as pluripotency genes and matrix metalloproteinases), only if the level of rigidity was less than ~100 Pa^76^. We used this finding to design grafts with a soft layer (~100 Pa or less) in which to embed organoids of BTSCs and with more rigid layers (~700 Pa) of hyaluronan hydrogels in the backing to form a barrier to migration in directions other than the target tissue as well as ones with intermediate levels of rigidity (~200–300 Pa) to minimize adhesions.

Macro-scale rheological properties of hyaluronan hydrogels were determined using a stress-controlled cone-and-plate rheometer (TA Instruments, AR-G2, 40 mm cone diameter, 1° angle). Gels actively polymerized on the rheometer while oscillating at 1 rad/s frequency and 0.6 Pa stress amplitude with the modulus monitored continuously to query for sufficient completion of the cross-linking reactions. Once equilibrated, the hydrogels were subjected to an oscillatory frequency sweep (stress amplitude: 0.6 Pa, frequency range: 0.01–100 Hz).

Preparation of cells was done from extrahepatic biliary tree tissue (gall bladder, common duct, hepatic ducts) obtained from pigs [or from human tissue]. For the tissues used to generate organoids of normal cells, tissues were pounded with a sterilized, stainless-steel mallet to eliminate the parenchymal cells, carefully keeping the linkage of the intra-hepatic and extrahepatic bile ducts. The biliary tree was then washed with the “cell wash” buffer comprised of a sterile, serum-free basal medium supplemented with antibiotics, 0.1% serum albumin, and 1 nM selenium (10^–9^ M). It was then mechanically dissociated with crossed scalpels, and the aggregates enzymatically dispersed into a cell suspension in RPMI-1640 supplemented with 0.1% bovine serum albumin (BSA) [or for human tissues, human serum albumin, HAS], 1 nM selenium, 300 U/ml type IV collagenase, 0.3 mg/ml deoxyribonuclease (DNAse) and antibiotics. Digestion was done at 32 °C with frequent agitation for 30–60 min. Most tissues required two rounds of digestions followed by centrifugation at 1100 rpm at 4 °C. Cell pellets were combined and re-suspended in cell wash. The cell suspension was centrifuged at 30×*g* for 5 min at 4 °C to remove red blood cells. The cell pellets were again re-suspended in cell wash and filtered through a 40 µm nylon cell strainer (Becton Dickenson Falcon #352340) with fresh cell wash. The cell numbers were determined, and viability was assessed using Trypan Blue. Cell viability above 90–95% was observed routinely.

Formation of organoids was done from cell suspensions added to multiwell, flat-bottom cell culture plates (Corning #353043) in serum-free Kubota’s medium and incubated for ~an hour at 37 °C to facilitate attachment of mature mesenchymal cells (e.g. mature stroma, mature stellate cells). Mature mesenchymal cells attached to the dishes within ~15 min even though the medium was serum-free. The immature cells remaining in suspension and were transferred to another dish and again incubated for up to an hour. This was repeated several times to ensure the depletion of a significant fraction of the mature mesenchymal cells. After depletion of mature mesenchymal cells, the remaining floating cells were seeded at ~2 × 10^5^ cells per well in serum-free Kubota’s medium in Corning’s ultralow attachment dishes (Corning #3471) and were incubated overnight at 37 °C in a CO_2_ incubator. Organoids comprised of the biliary tree stem cells (BTSCs) partnered with early lineage stage mesenchymal cells (ELMSCs) formed overnight. The ELMSCs were found by their expression of specific surface antigens to be angioblasts (CD117^+^, CD31^−^, VEGFr^+^) and precursors to endothelia (CD31^+^, VEGFr^+^) and to stellate cells (ICAM-1^+^, CD146^+^). More extensive characterizations of these ELSMCs were done, and the findings have been published previously^77,78^. The organoid cultures survived for weeks in Kubota’s medium, especially if the medium was supplemented (0.1%) with soluble forms of hyaluronans (Sigma).

The cells could also be cryopreserved as described below. From each gram of pig biliary tree tissue, we obtained ~1.5 × 10^7^ cells. We used ~3–6 × 10^5^ cells per well of a 6-well, ultra-low attachment plate and incubated in the serum-free Kubota’s medium. The cells produced, on average, 6000–20,000 small organoids (~50–100 cells/organoid/well). For the grafts, we used at least 100,000 organoids (>10^7^ cells). Depending on the size of the backing, we were able to increase the number of organoids in the grafts up to >10^8^ organoids (i.e. ~10^9^ cells total) or more embedded in ~1 ml of the soft hyaluronan hydrogel on a 3 cm × 4.5 cm backing.

Cryopreservation of stem/progenitors was most successfully done by cryopreservation of isolated cells or small cell aggregates that had been subjected to the panning procedures to minimize the numbers of mature mesenchymal cells. One can cryopreserve the mix of stem/progenitors and early lineage stage mesenchymal cells in Cryostor10, an isotonic cryopreservation buffer containing antifreeze factors, dextran, and DMSO (Bioliife, Seattle, WA). The viability of the cells was improved further with supplementation with 0.1% HAs (Sigma #52747)^71,72^. The highest viability of the organoids was when prepared from freshly thawed, cryopreserved cell suspensions; lower viabilities were observed with cryopreserved, fully formed organoids. Cryopreservation was done using CryoMed™ Controlled-Rate Freezers. The viability on thawing was greater than 90% for those cryopreserved as a cell suspension and then, upon thawing, used to form organoids^71,72^.

Patch grafts are novel methods established rapidly to transplant large numbers (e.g. >10^8^th) of organoids into solid organs and to have the organoids fully integrate within the organs within a week and mature into adult cells within another week. Further details of the logic, strategies, and methods for patch grafting of organoids into solid organs are given in our prior publications^54,55^. Grafts were formed using a backing, Contour Seri-silk (Sofregen, Medford, MA), onto which were placed the stem/progenitor organoids embedded in soft hyaluronan hydrogels (~50–100 Pa). These were readily prepared ahead of time and maintained in a culture dish in an incubator overnight. The lack of success with attempts to cryopreserve organoids within soft hydrogels meant that embedding the organoids in the soft hydrogel had to be done just prior to surgery to attach the grafts.

*Update*: We have learned that Contour Seri-silk is no longer available from the manufacturer. Amnion-based matrix materials, such as ones from Vivex Biologics (Miami, FL), should provide an appropriate substitute and is an FDA-approved implant material for clinical or non-clinical purposes. These should provide adequate mechanical support for the grafts and are hypothesized to be reasonably neutral in effects on donor organoids; this is important to ensure that the organoids retain their stem cell traits enabling them to express matrix metalloproteinases (MMPs) required for engraftment and migration^54^. Clinical uses of such amnion-derived matrices have been discussed by others in a recent review^79^. It is assumed that the more rigid hyaluronan hydrogel (~700 Pa) will not be required given the complex matrix chemistry within the amnion that should serve as the barrier. However, it is hypothesized that after tethering to the target site, an amnion matrix barrier might still require coating with hyaluronans on both sides and with the hyaluronan prepared with serum-free Kubota’s Medium and at an intermediate level of rigidity (~200–300 Pa). The coating on the serosal side at the time of surgery should minimize adhesions to the neighboring tissues.

For necropsy procedures, all animals were humanely euthanized at the designated time point by sedation with Ketamine/Xylazine, and isofluorane anesthesia, followed by an intravenous injection of a lethal dose of sodium pentobarbital. Upon confirmation of death, the carcass was carefully dissected, and the target organs were removed, and placed in chilled Kubota’s Medium for transportation to the lab. In addition to the liver, the lungs, heart, kidney, and spleen were collected and fixed in 10% neutral formalin.

Histology was done on portions of the freshly dissected tissues from the pigs or the grafts on the liver or pancreas were fixed with 4% paraformaldehyde (PFA) and then paraffin-embedded. Five-micron sections were cut and stained with hematoxylin-eosin for routine histological analyses. The tissues obtained from neonatal pigs were prepared also as samples (liver, pancreas, biliary tree and duodenum) in large paraffin blocks to facilitate analyses of the connections throughout the biliary tree and pancreatic duct system.

Immunohistochemistry (IHC) was used for analyses of various markers and traits. For immunofluorescent staining, 5 µm frozen sections or cultured cells were fixed with 4% paraformaldehyde (PFA) for 20 min at room temperature, rinsed with PBS, blocking with 10% goat serum in PBS for 2 h, and rinsed. Fixed cells were incubated with primary antibodies at 4 °C for 14 h, washed, incubated for 1 h with labeled isotype-specific secondary antibodies, washed, and counterstained with 4´,6-diamidino-2-phenylindole (DAPI) for visualization of cell nuclei and viewed using Leica DMIRB inverted microscope (Leica, Houston, TX) or a Zeiss ApoTome Axiovert 200 M (Carl Zeiss Inc, Thornwood, NY).

For IHC, the tissues were fixed in 4% PFA overnight and stored in 70% ethanol. They were embedded in paraffin and cut into 5 μm sections. After deparaffinization, antigen retrieval was performed with sodium citrate buffer (pH 6.0) or ethylenediaminetetraacetic acid (EDTA) buffer (pH 8.0) in a steamer for 20 min. Endogenous peroxidases were blocked by incubation for 15 min in 3% H_2_O_2_. Sections were incubated for 30 min at room temperature with ImmPRESS peroxidase staining kits and 3,3’-diaminobenzidine substrates (Vector Laboratories). Sections were counterstained with hematoxylin. Antibodies used are listed in Supplementary Table 1.

Purification of cell populations for genetic studies was important for various analyses. The purity of the subpopulations being isolated was dictated by a combination of strategies for the isolation of the cells. The adult hepatocytes were freshly isolated from neonatal porcine livers versus from neonatal, pediatric, and adult human livers by standard collagenase digestion and percoll fractionation^80^. The isolated adult hepatocyte fractions (those with average diameters above 17 µm) were rinsed in RPMI1640 supplemented with 2% serum to inactivate the enzymes used in isolation of the cells; then treated with multiple rounds of rinsing with serum-free RPMI 1640 medium; and finally, snap frozen using liquid nitrogen. The cells were not cultured.

The subpopulations of stem/progenitors, both human and porcine ones, were prepared from freshly isolated cells from the fetal or neonatal biliary tree (biliary tree stem cells), from fetal or neonatal livers (hepatic stem cells and hepatoblasts), or fetal or neonatal pancreases (pancreatic stem cells and ductal progenitors); purified by a combination of immunoselection for surface antigens, enabling the isolation of separate cellular sub-populations^39,41,50,54^. Supplementary Table 5 summarizes the markers of the subpopulations that have been characterized extensively, especially in past studies^31–33,38–43,49,50^.

The immunoselected cells were suspended in serum-free Kubota’s Medium^81^, designed for endodermal stem cells, and seeded onto low attachment culture dishes for 6–12 h enabling the formation of organoids. The organoids that formed in that 6–12 h comprised the endodermal stem cells partnered with early lineage stage mesenchymal cells: angioblasts and precursors to endothelial and stellate cells (note: the mature mesenchymal cells were minimized by the immunoselection process as indicated by the depletion of cells for surface antigens for the mature hemopoietic and mesenchymal cells).

RNA-sequencing and gene expression analysis included RNA purified using Qiagen RNeasy Kit from the porcine (or human) adult liver, pancreas, gallbladder, and biliary tree tissue and from isolated cell suspensions of hepatocytes (AHeps), hepatic stem cells (HpSCs), biliary tree stem cells (BTSCs), each from three different donors for human cells, and five different donors for pig cells (Supplementary Tables 6 and 7). RNA-seq data collection and quality control analyses were specifically depicted in our previous study^52^. The LIMMA (version 3.50.1) pipeline was used to identify differentially expressed genes (DEGs). Gene expression profiles were compared using Pearson’s and Spearman’s correlation analyses. Principal component analysis (PCA) and hierarchical clustering were performed in R (R version 4.1.0).

Quantitative reverse transcription and polymerase chain reaction were done using total RNA from cells. Total RNA was extracted from the cells using Trizol (Invitrogen, Carlsbad, CA). First-strand cDNA synthesized using the Prime script 1st strand cDNA synthesis kit (Takara, Otsu, Japan) was used as a template for PCR amplification. Quantitative analyses of mRNA levels were performed using Faststart Universal Probe Master (R oche Diagnostics, Mannheim, Germany) with ABI PRISM 7900HT Sequence Detection System (Applied Biosystems, Foster City, CA). Primers were designed with the Universal Probe Library Assay Design Center (Roche Applied Science). Primer sequences are listed in Supplementary Table 4a. The primers were annealed at 50 °C for 2 min and 95 °C for 10 min, followed by 40 cycles of 95 °C (15 s) and 60 °C (1 min). Expression of glyceraldehyde-3-phosphate dehydrogenase (GAPDH) was used generally as a standard.

### Statistical analysis

Statistically significant differences between samples are calculated by using Student’s 2-tailed *t*-test and results are presented as the mean ± SD. *P* values of <0.05 were considered statistically significant.

### Reporting summary

Further information on research design is available in the Nature Portfolio Reporting Summary linked to this article.

### Supplementary information

supplementary information

Reporting summary

Supplementary Movie 1. Porcine intrahepatic and extrahepatic biliary tree.

Supplementary Movie 2. Common duct of the porcine extrahepatic biliary tree.

Supplementary Movie 3. Scan of Porcine pancreatic biliary tree.

Supplementary Movie 4. Scan of Porcine pancreatic biliary tree-2.

Supplementary Movie 5 Animation of pig vs dog embryo development.

Supplementary Movie 6 Animation of human biliary tree.

## Data Availability

The data that support the findings of this study are openly available in Gene Expression Omnibus (GEO) with the GEO accession number GSE73114 for the human RNA-seq data and GSE229270 for the porcine dataset. All data used in the study were collected and normalized to a newly constructed user-friendly website using shinyapp in R (https://wangxc.shinyapps.io/BTSCs_FLCs). All data and materials are available to all interested parties. No data were (or are) kept secret.

## Code availability

All analyses were performed in R (R version 4.1.0). Any questions by interested researchers related to the analytic process of the sequencing data should contact the corresponding authors with respect to genetic signature studies and bioinformatics, Z.H. or P.S.
