# Publisher Correction: A postnatal network of co-hepato/pancreatic stem/progenitors in the biliary trees of pigs and humans

**DOI:** 10.1038/s41536-023-00323-1

**Published:** 2023-09-06

**Authors:** Wencheng Zhang, Xicheng Wang, Giacomo Lanzoni, Eliane Wauthier, Sean Simpson, Jennifer Ashley Ezzell, Amanda Allen, Carolyn Suitt, Jonah Krolik, Alexander Jhirad, Juan Dominguez-Bendala, Vincenzo Cardinale, Domenico Alvaro, Diletta Overi, Eugenio Gaudio, Praveen Sethupathy, Guido Carpino, Christopher Adin, Jorge A Piedrahita, Kyle Mathews, Zhiying He, Lola McAdams Reid

**Affiliations:** 1 Department of Cell Biology and Physiology, University of North Carolina (UNC) School of Medicine, Chapel Hill, NC 27599 USA; 2 Institute for Regenerative Medicine, Shanghai East Hospital, School of Life Sciences and Technology, Tongji University School of Medicine, 200123 Shanghai, China; 3 Shanghai Engineering Research Center of Stem Cells Translational Medicine, 200335 Shanghai, China; 4 Shanghai Institute of Stem Cell Research and Clinical Translation, 200120 Shanghai, China; 5 Diabetes Research Institute, Leonard Miller School of Medicine, 1450 N.W. 10th Avenue, Miami, FL 33136 USA; 6 Department of Molecular Biomedical Sciences, North Carolina State University (NCSU) College of Veterinary Medicine, Raleigh, NC 27606 USA; 7 Comparative Medicine Institute, NCSU, Raleigh, NC 27606 USA; 8 Center for Gastrointestinal Biology and Disease (CGIBD), UNC School of Medicine, Chapel Hill, NC 27599 USA; 9 Department of Medico-Surgical Sciences and Biotechnologies, Sapienza University, Rome, Latina 04100 Italy; 10 Department of Translational and Precision Medicine, Sapienza University, Rome, 00185 Italy; 11 Department of Anatomical, Histological, Forensic Medicine and Orthopedics Sciences, Sapienza University, Rome, 00161 Italy; 12 Department of Biomedical Sciences, Cornell University College of Veterinary Medicine, Ithaca, NY 14853 USA; 13 Department of Clinical Sciences, Soft Tissue and Oncologic Surgery Service, College of Veterinary Medicine, NCSU, Raleigh, NC 27606 USA; 14 Program in Molecular Biology and Biotechnology, UNC School of Medicine, Chapel Hill, NC 27599 USA; 15 Present Address: Department of Small Animal Clinical Sciences, University of Florida College of Veterinary Medicine, Gainesville, FL 32608 USA

**Keywords:** Regeneration, Adult stem cells

Correction to: *npj Regenerative Medicine* 10.1038/s41536-023-00303-5, published online 01 August 2023

In this article the corresponding author email addresses were in advertently missed to Praveen Sethupathy, Guido Carpino, Christopher Adin, Jorge Piedrahita, Kyle Mathews, Zhiying He. The original article has been corrected.

