# Supplementary material for: A postnatal network of co-hepato/pancreatic stem/progenitors in the biliary trees of pigs and humans
Source: NPJ Regen Med. 2023 Aug 1;8:40. doi: 10.1038/s41536-023-00303-5 (PMC10394089; doi:10.1038/s41536-023-00303-5)
Supplement: Supplementary file 1 — supplementary information [file 41536_2023_303_MOESM1_ESM.pdf]

ONLINE SUPPLEMENT FIGURES

https://doi.org/10.1038/s41536-023-00303-5; URL: 10.1038/s41536-023-00303-5

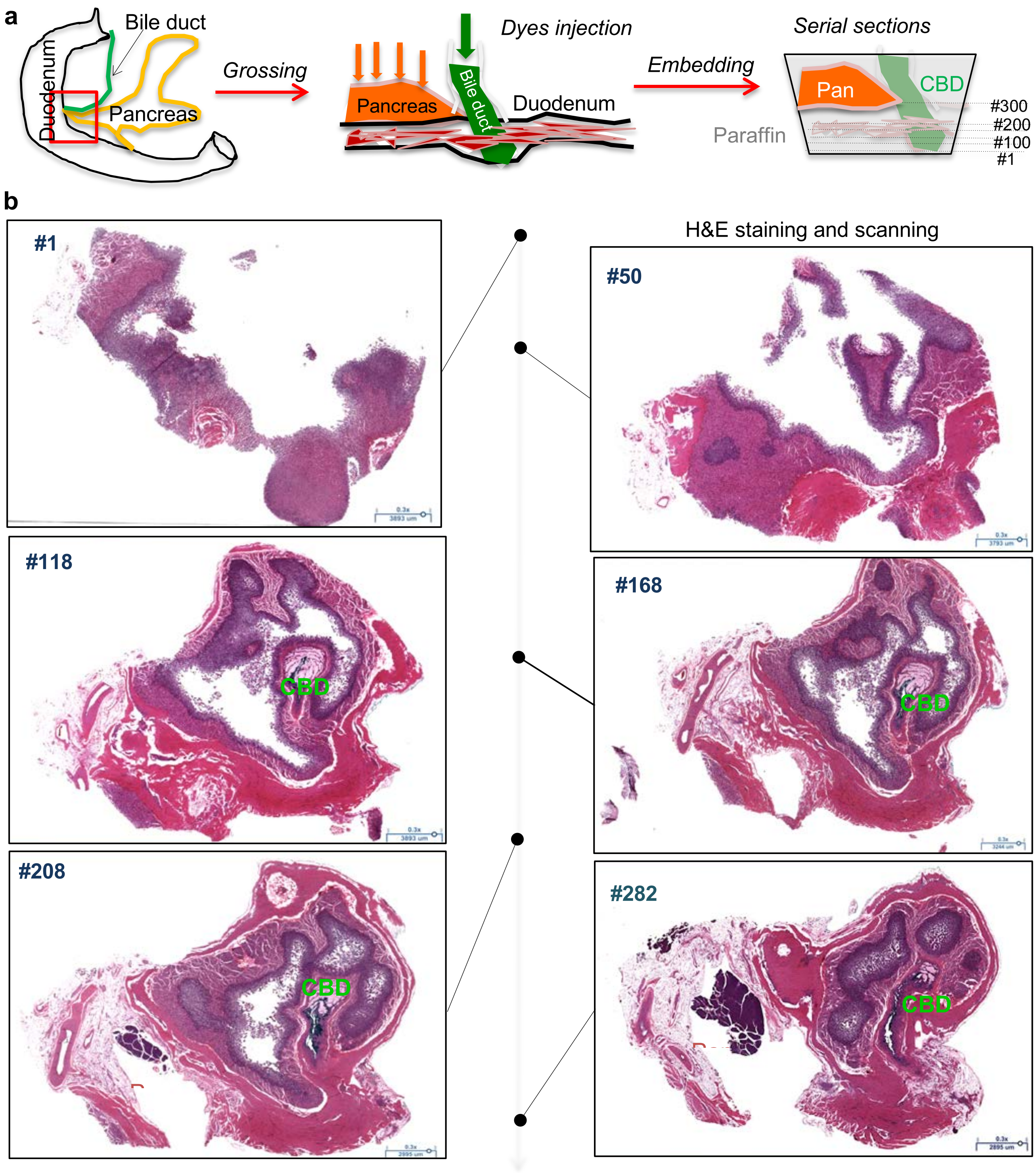

Supplementary Figure 1. Serial sections of common bile duct and the associated pancreatic duct in 4-week-old piglets confirmed findings in past studies that there is no hepato-pancreatic common duct in postnatal pigs.

a. A simple graphic was used to show the procedure of tissue grossing, processing, embedding and serial section. A total of 300 sections were collected starting from the inside of the duodenum's mucosa to the surface of the outside of the muscular layer of the duodenum.

b. H&E staining of serial sections with a thickness of 5 μm. The stained section was scanned for every single slide from slides #1 to #300. The opening of the entrance of bile duct appeared by #50. The injected green tissue dye can be distinguished by slides # 118. An enlarged lumen of common bile duct with green tissue dye can be found from #168 to #208. There were few mucosae shown when sections were taken from the layer of #282.

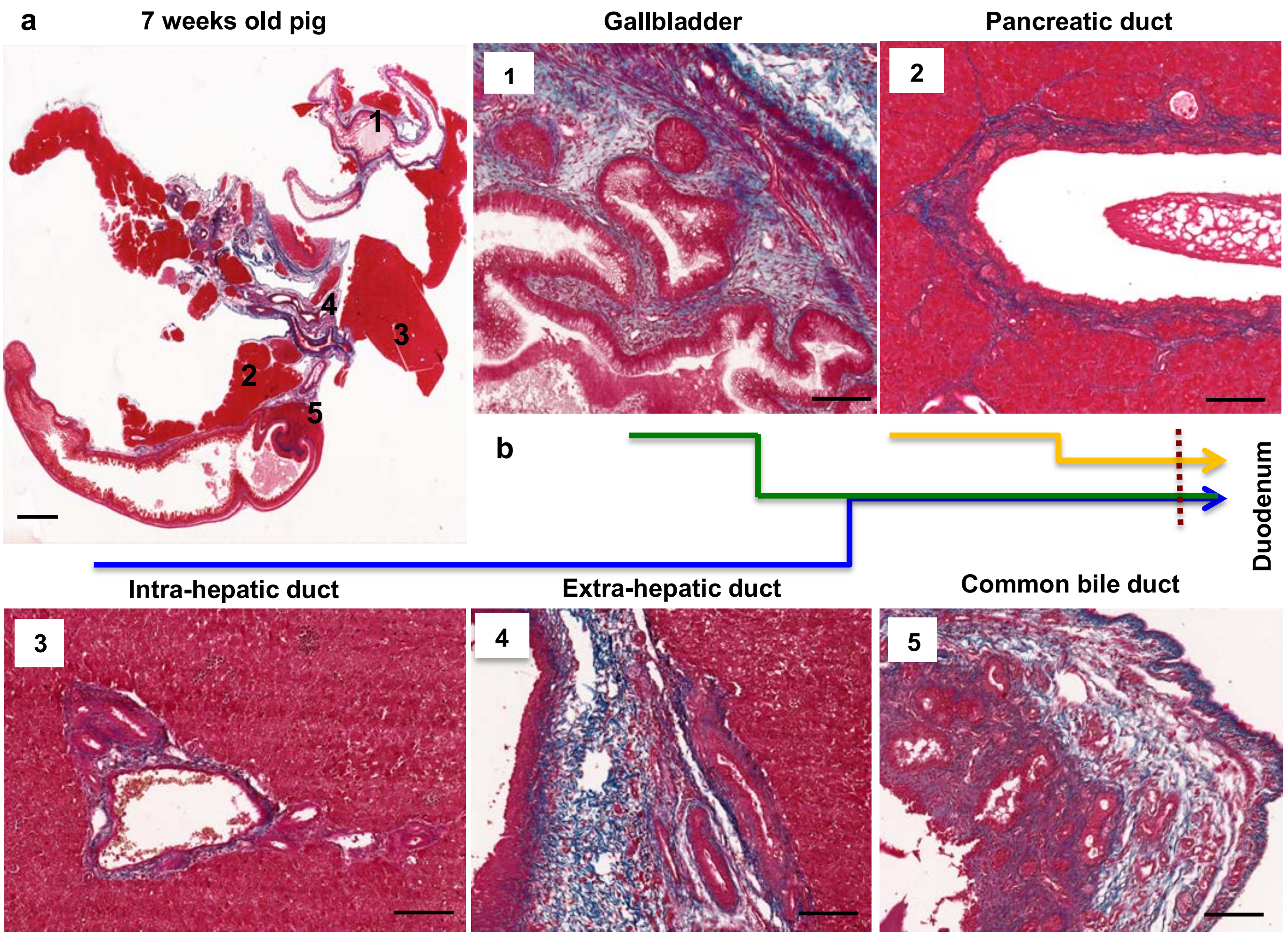

Supplementary Figure 2. Histology for 7-week-old pig liver, biliary tree, and duodenum

Masson's trichrome staining of sections from the 7-week-old piglet embedded in the large FFPE paraffin block. Compared to newborn piglets, the pancreas of the 7-week-old piglet is dramatically larger. (**a**) Magnification 0.6X. The gallbladder has more villi in the mucosa compared to that in the newborn (**a, 1**). Intramural glands in the walls of the pancreatic duct (**a, 2**). When the intrahepatic duct (**a, 3**), and extrahepatic duct (**a, 4**) and the common bile duct are close to the duodenum (**a, 5**) there are also sites enriched for PBGs. A simple flow chart along the histology graphs of each duct, shows the hepatic-biliary tree and the pancreatic biliary tree merge into the duodenum separately. **b**, **Blue**=intra-/extrahepatic duct; **Green**=gallbladder cystic duct; **Yellow**=pancreatic duct. Scale bar of the enblock in **a** is 4 mm, for all images in **b** are 100 μm.

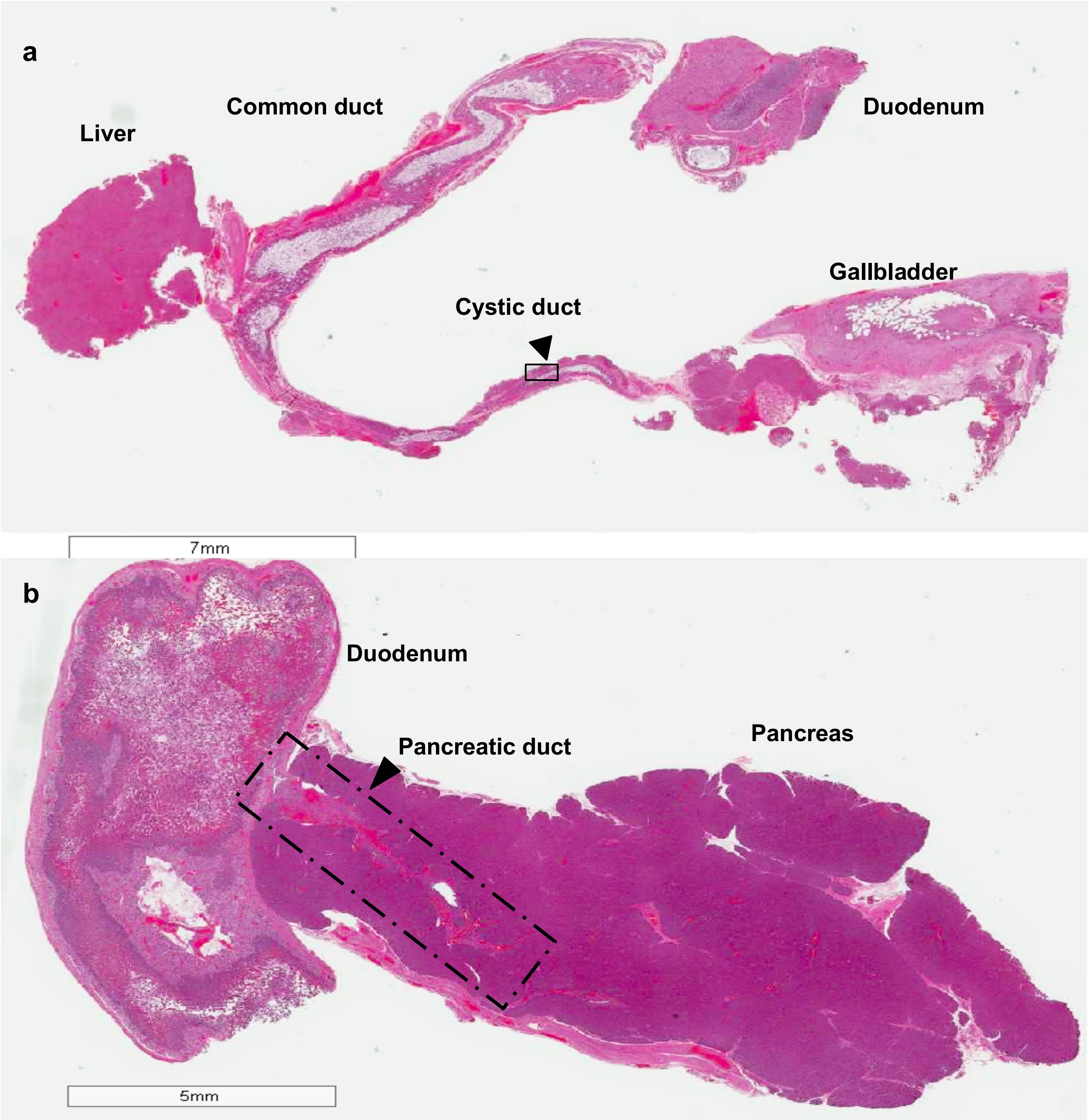

Supplementary Figure 3. Histology of the biliary tree and its connections with the liver and pancreas

These indicate the ductal structures for 7-week-old piglets (scale bar = 7 mm). A section of the pancreas and the duodenum is used to indicate the structure of the junction and connections between the biliary tree and the pancreas for weanling piglets (scale bar = 5 mm).

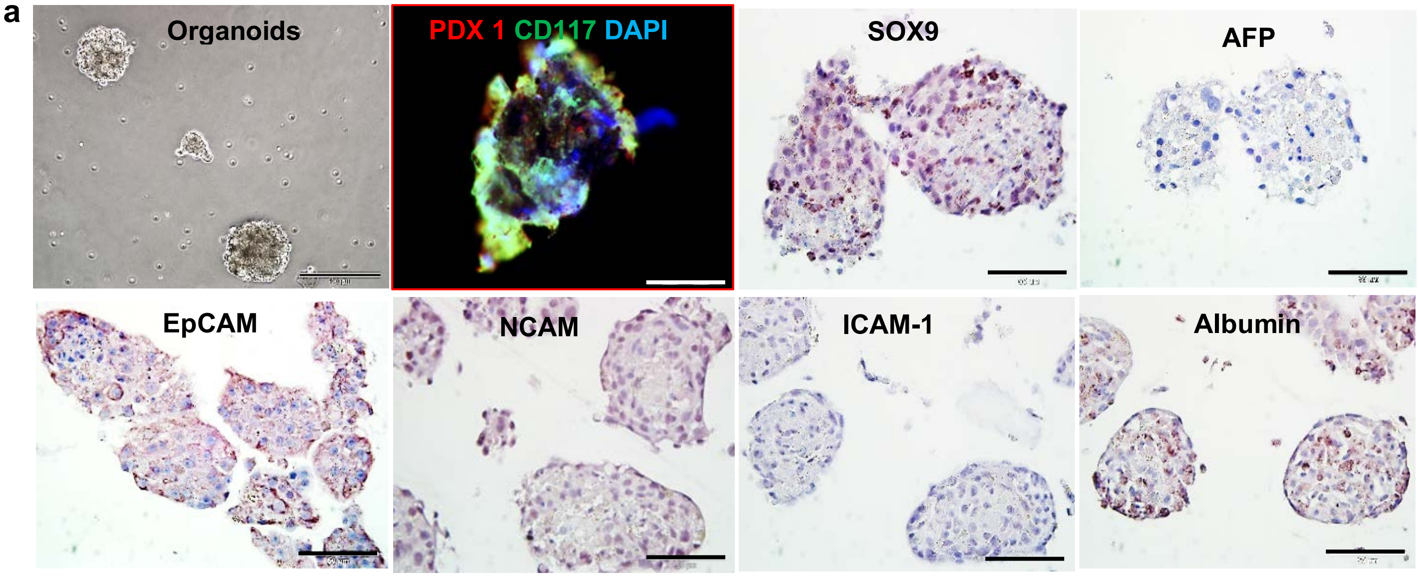

Supplementary Figure 4. ***Ex Vivo*** **Differentiation potential of pBTSC/ELSMC organoids**

Wholly defined, serum-free culture conditions were used to establish organoids from isolated porcine biliary tree cells. These were subjected to distinct serum-free conditions for stemness versus for lineage restriction towards hepatic versus pancreatic fates. The details of the different conditions are provided in **Supplementary Table 3**. Thus, they were maintained either in serum-free Kubota's Medium for maintenance of stemness traits or in a serum-free hormonally defined medium (HDM) tailored to optimized differentiation to liver versus pancreas[1,2].

a. For maintenance as organoids of stem/progenitors, they were maintained in serum-free Kubota's Medium supplemented with 0.1% hyaluronan. These conditions are devoid of growth factors and of hormones other than insulin and transferrin/Fe. The organoids remained indefinitely as stem/progenitors of pBTSCs partnered with early lineage stage mesenchymal cells, angioblasts and their descendants, precursors to endothelia and to stellate cells. The pBTSCs expressed SOX2, SOX9, NCAM, SOX17 and PDX1. They did not express mature hepatic or pancreatic markers (e.g. albumin, P450s, CFTR, insulin, other islet hormones, amylase), nor early or intermediate lineage stage hepatic or pancreatic traits (e.g. alpha-fetoprotein, ICAM-1, MUC 6). The scale bar for the phase contrast image is 100 μm; that for the IF and IHC staining are 50 μm.

b

Hepatic differentiation

Conditions as specified in **Supplementary Table 3** and with substratum of type IV collagen

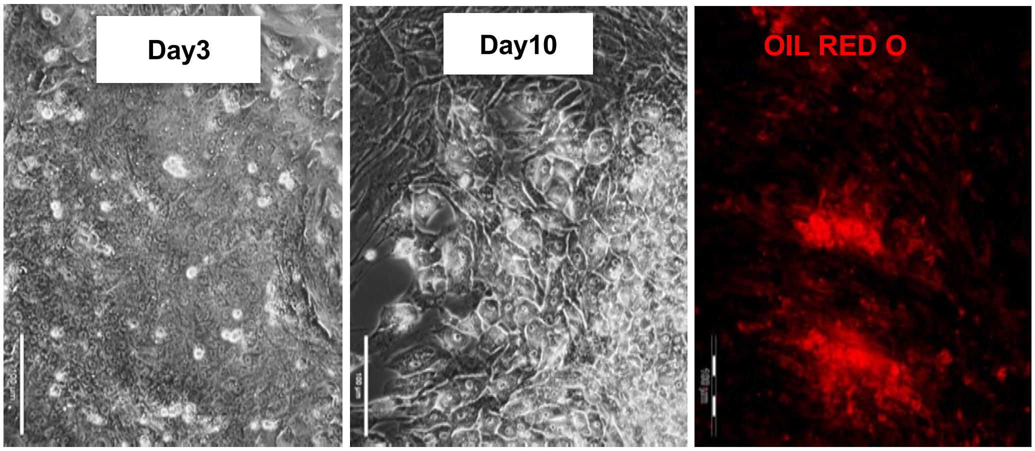

c

Pancreatic differentiation

Conditions as specified in **Supplementary Table 3** and with medium supplemented with type IV collagen

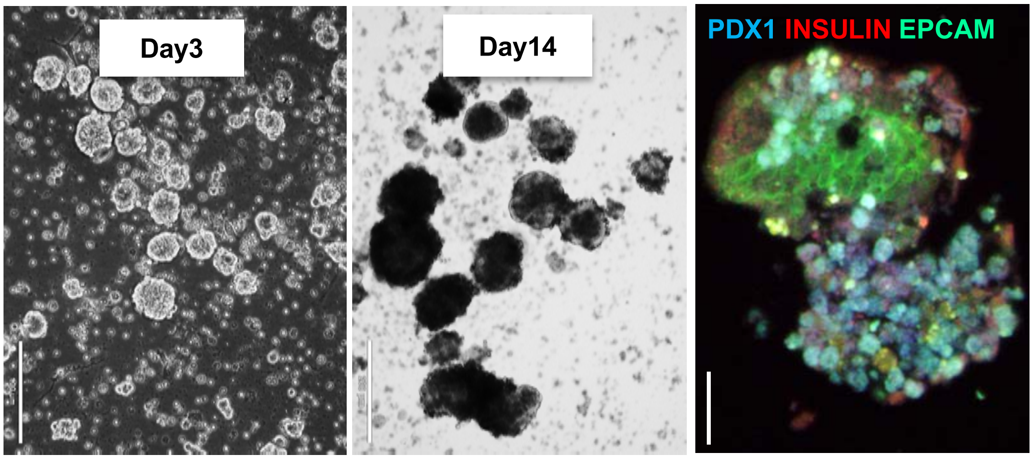

b. For lineage restriction towards hepatic fates, Kubota's Medium was supplemented with higher calcium levels (0.6 mM), copper (10^{-10} M), and the hormones and growth factors (glucocorticoids, HGF, EGF, and Oncostatin M) and at concentrations as noted in **Supplementary Table 3**. For rapid and optimal differentiation, the organoids were plated onto substrata of type IV collagen and laminin, mixed at a 1:1 ratio. Within 24-48 hours, the organoids converted to monolayer cultures showing classic parenchymal morphology. Shown after one week under the differentiation conditions is their expression of lipids (oil red O staining). They also expressed other mature hepatic traits (not shown) that included albumin, P450s, and CFTR. There are far more extensive characterizations of the porcine organoids under these conditions *ex vivo* provided in a separate report and that were consistent with previous findings with human cells[3]. Scale bar = 100 μm for all images.

c. For lineage restriction towards pancreatic islet fates, Kubota's Medium was prepared without glucocorticoids and further supplemented with 0.6 mM calcium, 1% B27, 0.1 mM ascorbic acid, 0.25 μM cyclopamine, 1 μM retinoic acid, 20 ng/ml of FGF-7 for 4 days, and then changed to one supplemented with 50 ng/ml Exendin-4 and 20 ng/ml of HGF for 6 more days of induction. Though the pancreatic islet fates were achieved in these studies without the addition of matrix components, more rapid and complete differentiation to islet fates occurred with supplementation of the medium with type IV collagen and laminin (ratio of 1:1) in data not shown. Within less than a week, the cells transitioned to islets and acquired expression of insulin, glucagon, and other islet hormones. These findings were consistent with our prior findings with lineage restriction of human cells to islet fates [2]. Scale bar for Day 3 = 100 μm, for Day 14 = 200 μm, for the IF staining = 20 μm.

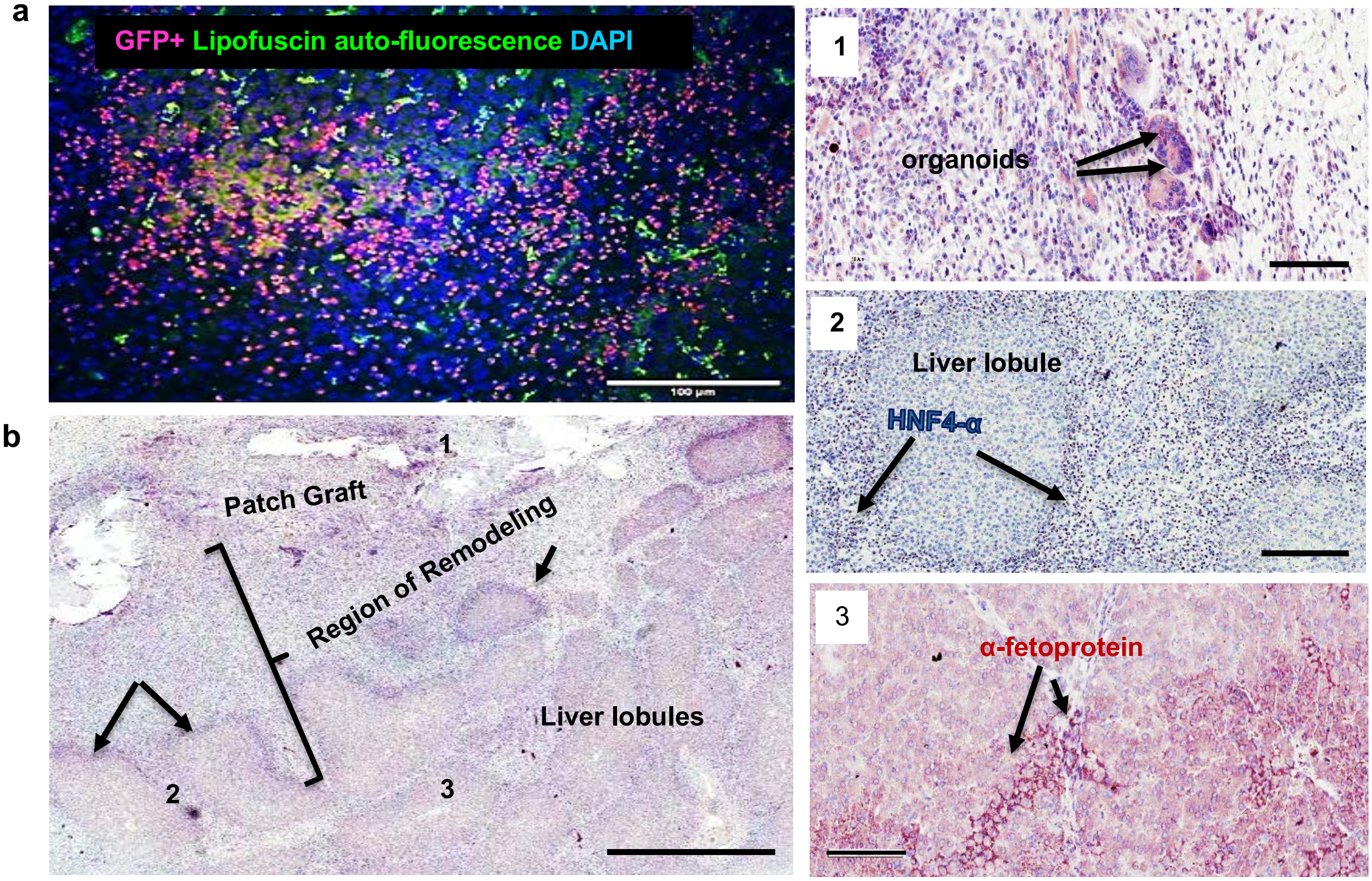

Supplementary Figure 5. *In Vivo* Differentiation potential of organoids of porcine GFP+ BTSCs in patch grafts tethered to the liver versus pancreas of wild type pigs.

a. Patch graft of GFP^{+} biliary tree stem/progenitor organoids on the liver of a wild type piglet. Graft site at one week post-transplantation. The liver section was stained with an antibody to GFP and that was linked to Novored. Host nuclei are blue (4,6-Diamidino-2-phenylindole; DAPI). The GFP^{+} donor cells have pink nuclei (merger of Novored and the DAPI). Even at a week, the donor cells had engrafted throughout the liver. The spring green color indicates stellate cells' vitamin A autofluorescence; the forest green indicates parenchymal cells due to their autofluorescing lipofuscins. Scale bar =100 µm.

b. Patch graft of GFP^{+} biliary tree stem/progenitor organoids on the liver of a wild type piglet. Graft site at one week showing loss of Glisson`s capsule and of altered hepatic histological features. Scale bar = 500 µm. In sites (1) (2), and (3), there are enlargements, shown on the right, and indicating that the engrafting organoids in the patch (1), and engrafted cells in the liver parenchyma are acquiring hepatic traits such as the hepatic transcription factor, HNF4α (2), and a-fetoprotein (AFP) (3). Scale bars for **b1** and **b3** = 100 µm, **b2** = 200 µm.

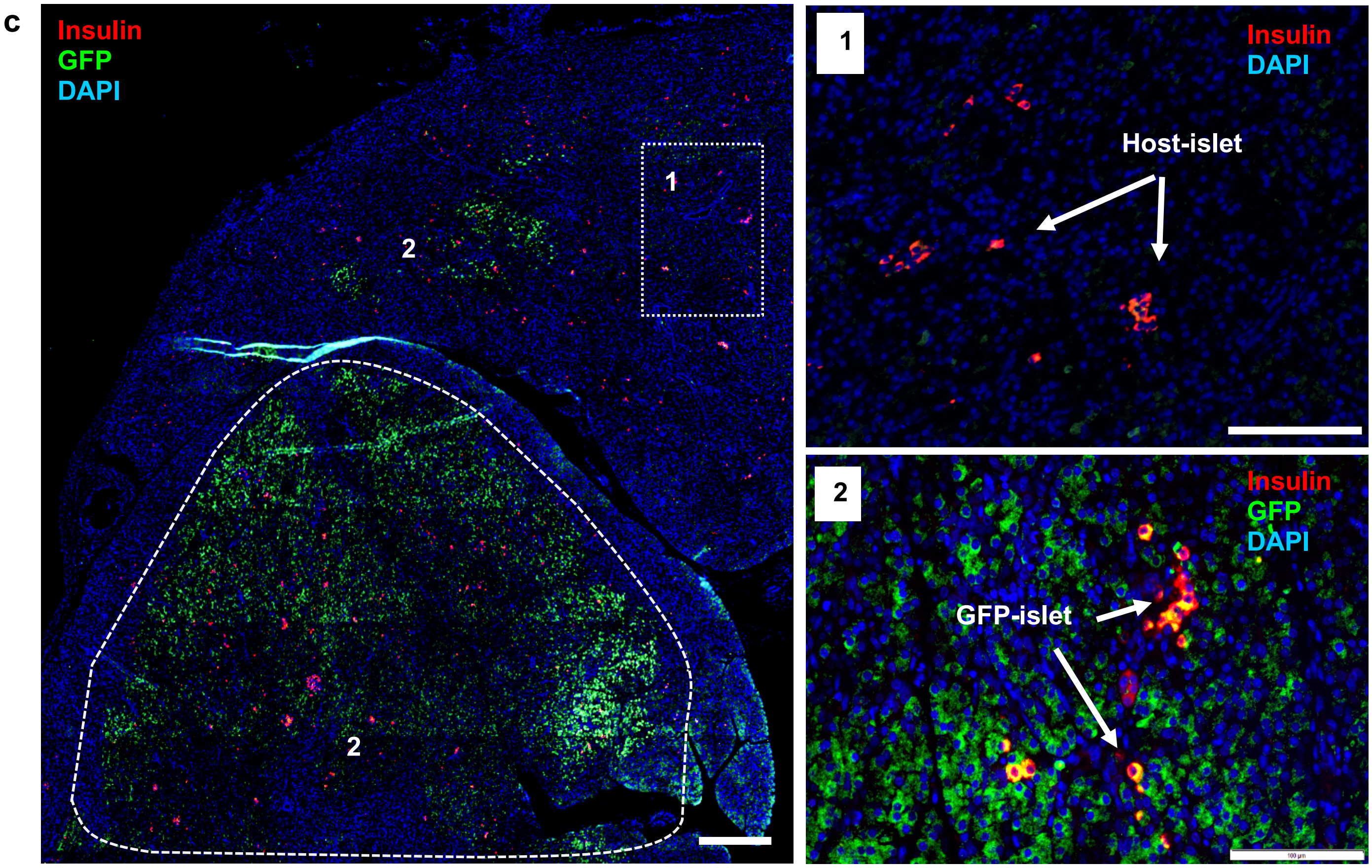

c. Patch graft of GFP+ biliary tree stem/progenitor organoids on the pancreas of a wild type piglet. Within a week GFP+ donor cells had engrafted and migrated throughout much of the pancreas. The section was stained with DAPI for the nuclei. Scale bar = 2 mm. This enabled one to identify donor acinar cells (GFP+); host acinar cells (DAPI+); host islets (1, red) and donor islets (2, yellow/orange color from merger of GFP and red fluoroprobes). Scale bar = 50 μm for both **c1** and **c2**.

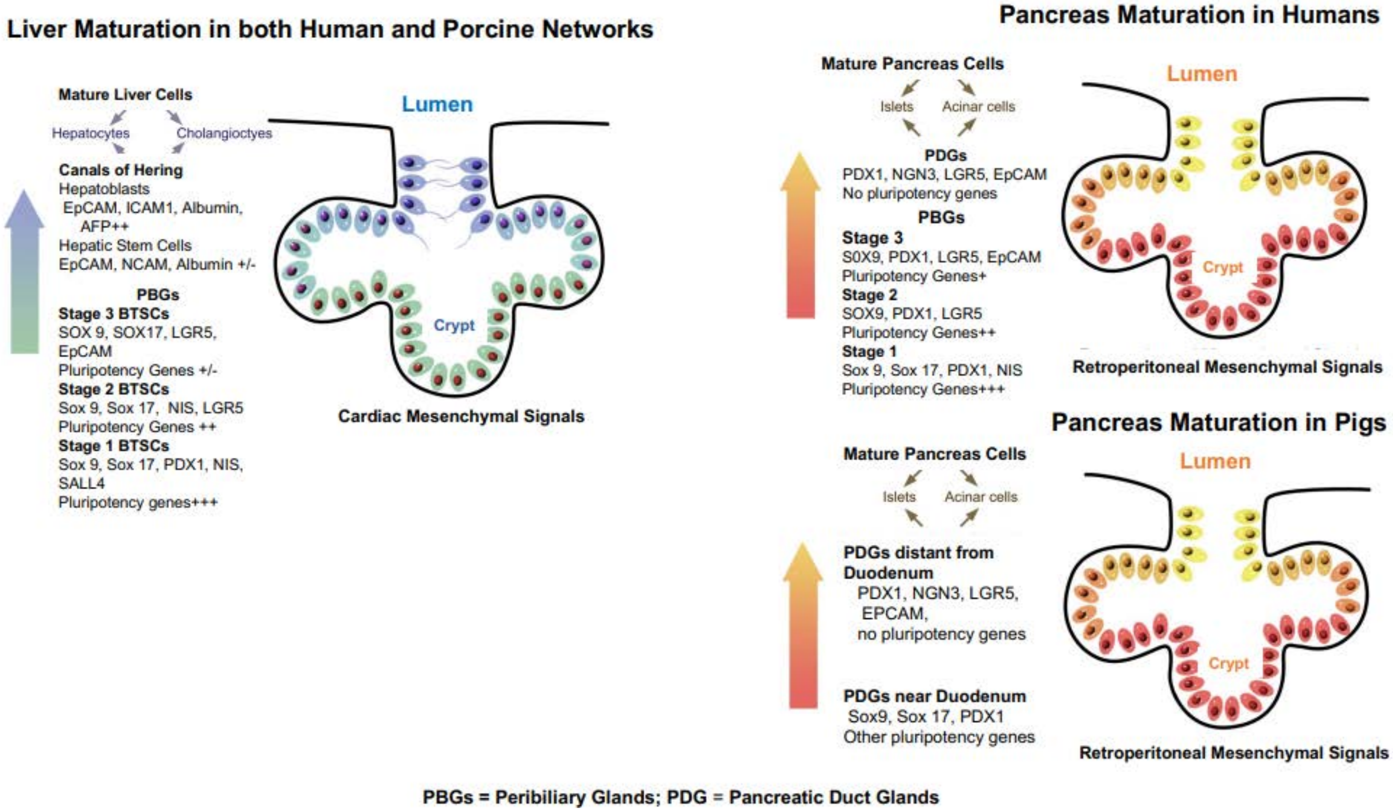

Supplementary Figure 6. Comparison of human versus porcine networks in liver and pancreas. This figure is a modified version of one presented in a book chapter on the human endodermal stem cell networks in the biliary tree[4] and with additional input from information in a review[5] and in a recent journal article[6]. Porcine endodermal stem cell maturational lineages in the liver are similar to those in human livers but are distinct to those in the pancreas.

Liver: Porcine endodermal stem cell maturational lineages in livers parallel those in human livers. The most primitive endodermal stem cells are located in the submucosa of the duodenum (Brunner's Glands) and in the presumptive crypts of the peribiliary glands (PBGs). These presumptive crypts are near the fibromuscular layer below the bottoms of the PBGs. The stem cells move along the sides of the walls of the PBGs and towards the lumens. In that process, they undergo maturation to adult fates achieved in the cells near to the lumens.

Pancreas: The maturational lineages for pancreas are distinct in humans versus pigs. In humans, the endodermal stem cells are found in the presumptive crypts of the hepato/pancreatic duct's PBGs. The connections also with Brunner's Gland's duodenal stem cells are assumed but not yet proven. Only committed progenitors are present in intrapancreatic ducts and in the pancreatic duct glands (PDGs). In pigs, there is no hepato/pancreatic common duct. Instead, the stem cells are found in the PDGs near to the duodenum. With increasing distance from the duodenum, only committed pancreatic progenitors are found in the PDGs.

ONLINE SUPPLEMENT TABLES

Supplementary Table 1. Key Resources: Antibodies						
**Antibody**	**Species Type**		**Manufacturer**	**Clone**	**Catalog#**	**Dilution for testing**
NANOG	Rb	IgG	Peprotech	D73G 4	500-P236	1/200(IHC-P, ICC-IF),
SOX2	Rb	IgG	Cell Signaling	D6D9	#3579	1/50(IHC)-400(ICC-IF)
OCT4	Rb	IgG	Cell Signaling	/	#2750	1/200(IHC)
EpCAM (C-terminal)	Rb	IgG	Abcam Origene	E144	ab32392	1/200 1/100(ICC-IF)
SOX17	Ms	IgG1	Abcam	3B10	ab84990	1/100(IHC-P) 1/20(ICC-IF)
SOX9	Rb	IgG	Chemicon	/	AB5535	1/500-2000, IHC-P 1/1000
Ki67	Rb	IgG1	Abcam	SP6	ab16667	1/100(ICC-IF)-100(IHC)
FOXA2	Rb	IgG	Abcam	/	ab40874	1/500
CXCR4	Rb	IgG	Abcam	/	ab2074	1/100(IHC-P)-500(ICC-IF)
RGS8	Rb	IgG	SantaCruz	/	sc-134552	1/50(IHC-ICC-IF)1/100(IHC-P)
RGS16	Rb	IgG	Applied Biological Materials	/	Y054685	1/50(IHC-ICC-IF)1/75(IHC-P)
AFP	Ms	IgG2a	SIGMA	C3	A-8452	1/100(ICC-IF) 1/500(IHC-P)
ASBT (SLC10A2)	Rb	IgG	Gift-Dawson P	SLC1 0AC	7789	1/1000 1/400(IF)
NIS (sodium iodide symporter)	Ms	IgG1	Abcam	SPM1 86	ab17795	1/50(IHC-P-IF)
CD44	Ms	IgG2a	Abcam	F10-44-2	ab6124	1/200(IHC-P) 1/100(ICC-IF)
PDX1	Gt	IgG	R&D	/	AF2419	1/50
Insulin	Rb	IgG	NOVUS	/	NBP1-50246	1/100
LGR5	Rb	IgG	Sigma	/	HPA012530	1/500IHC 1/350 ICC
Pig Albumin	Rb	IgG	Abcam	/	ab79960	IF;1/100
Insulin	Gp	IgG	Abcam	/	ab195956	IHC-P, IHC-Fr

Supplementary Table 2. Key Resources: Primers (qPCR)					
**Category**	**Name**	**Accession Number**	**Sequence (5' to 3')**	**Length (bp)**	**Tm (°C)**
Housekeeping gene	**GAPDH**	NM_001206359.1	**Forward**: ATCCTGGGCTACACTGAGGAC<br>**Reverse:** AAGTGGTCGTTGAGGGCAATG	473	60 °C
Pluripotency genes	**Nanog**	NM_001129971.1	**Forward**: TTCCTTCCTCCATGGATCTG<br>**Reverse**: ATCTGCTGGAGGCTGAGGTA	214	62°C
	**Sox2**	NM_001123197	**Forward**: GCCCTGCAGTACAACTCCAT<br>**Reverse**: GCTGATCATGTCCCGTAGGT	216	60 °C
	**Oct4**	JN633978.1	**Forward**: CGAAGCTGGACAAGGAGAAG<br>**Reverse**: GCTGAACACCTTCCCAAAGA	176	60 °C
Endoderm: primitive genes	**EpCAM**	NM_214419.1	**Forward**: ACCAGAGAATGCTATCCAGAAC<br>**Reverse**: CTCACTCGCTCCAAACAGG	314	53 °C
	**Lgr5**	NM_001315762.1	**Forward**: CCTTGGCCCTGAACAAAATA<br>**Reverse**: ATTTCTTTCCCAGGGAGTGG	110	60 °C
	**Bmi-1**	NM_001285971.1	**Forward**: TCATTGATGCCACAACCATT<br>**Reverse**: TGAAAAGCCCCGGAACTAAT	189	60 °C
Intestinal tract-related genes	**Muc2**	NC_010444.3	**Forward**: GGCTGCTCATTGAGAGGAGT<br>**Reverse**: ATGTTCCCGAACTCCAAGG	249	60 °C
	**CDX2**	NC_010453.4	**Forward**: AGAACCCCCAGGTCTCTGTCTT<br>**Reverse**: CAGTCCGAAACACTCCCTCACA	115	56°C
Hepatic parenchymal cells related genes	**AFP**	NM_214317.1	**Forward**: CGCGTTTCTGGTTGCTTACAC<br>**Reverse**: ACTTCTTGCTCTTGGCCTTGG	609	60
	**Albumin**	AY663543.1	**Forward**: AGTCTGCCAAGCTGCTGATA<br>**Reverse**: AGCCTTGGGAAATCTCTGGC	115	56

Supplementary Table 3. Serum-Free, wholly defined *ex vivo* conditions used for organoids

	Biliary Tree Stem/progenitor Organoids*	Lineage restriction to hepatocytes and cholangiocytes	Lineage restriction to pancreatic islets
**Basal Medium**	Any rich basal medium (e.g. RPMI 1640 or DME/F12) plus nicotinamide		
**Trace elements**	---	copper (10^{-10th} M)	
	selenium (10^{-9th}M), zinc (10^{-12th}M)		
**Calcium**	0.3 mM	0.6 mM	
**Insulin**	5 μg/ml		
**Transferrin/Fe**	5 μg/ml		
**Growth factors**	None	T3 (10^{-9}M), bFGF (20 ng/ml), HGF (20 ng/ml),	
		Oncostatin M (10 ng/ml) Galactose (2 gL), EGF (10 ng/ml); VEGF (20 ng/ml)	Cyclopamine (0.25 μM), retinoic acid (1 μM)
**Hormones**	None	Glucocorticoids (10^{-8th} M) Glucagon (7 μg/ml)	Exendin (50 ng/ml) FGF-7 (20 ng/ml)
**Lipids****	10 μg/ml high density lipoprotein plus a mixture purified free fatty acids bound to purified albumin		
**Matrix components** (***elicited rapid differentiation)	0.1% hyaluronans	A substratum of type IV collagen and laminin (ratio 1:1)	Supplementation of medium with type IV collagen and laminin (ratio 1:1)
**Oxygen levels**	2%	5%	

*The organoids were comprised of biliary tree stem cells partnered with angioblasts and their immediate descendants, precursors to endothelia and to stellate cells. In serum-free Kubota's Medium supplemented with 0.1% soluble hyaluronans, the organoids remained viable stably as stem/progenitors for weeks with medium changes every 1-2 days.**Details of the preparation of Kubota's Medium are as described previously[1].*** Matrix components conferred stability. Differentiation occurred with serum-free, hormonally defined medium (HDM) alone, but it happened more quickly (within 2-3 days) and more completely if the purified matrix components were also used.

Supplementary Table 4. Pigs used for Studies on Anatomy, Histology, Immunohistochemistry or Cell Culture			
**#**	**Age**	**Sex**	**Use of the Tissue and the Fixation buffer**
**PL-01**	9 weeks	male	Anatomical study: pancreas-liver-gallbladder-cystic duct-bile duct-duodenum
**PL-02**	1 week	male	Anatomical study: pancreas-liver-gallbladder-cystic duct-bile duct-duodenum
			4% PFA -70% ethanol before processing
**PL-03**	1 week	male	Anatomical study: pancreas-liver-gallbladder-cystic duct-bile duct-duodenum
			4% PFA -70% ethanol before processing
**PL-04**	7 weeks	male	Anatomical study: pancreas-liver-gallbladder-cystic duct-bile duct-duodenum
			4%PFA -70% ethanol before processing
**PL-05**	4 weeks	male	Samples were used for cell isolation
**PL-06**	3 weeks	male	Samples were used for cell isolation
**PL-07**	3 weeks	male	Samples were used for cell isolation
**PL-08**	1 week	male	Samples were used for Indian Blue Ink study
**PL-09**	12 weeks	female	Samples were used for cell isolation
**PL-10**	12 weeks	male	Anatomical study: pancreas-liver-gallbladder-cystic duct-bile duct-duodenum
			4% PFA 1 week-70% ethanol before processing
**PL-11**	1 week	male	Samples were used for cell isolation and cryopreservation
**PL-12**	9 weeks	male	Anatomical study and samples were used for cell isolation
**PL-13**	1 week	male	Samples were used for cell isolation
**PL-14**	1 week	male	Samples were used for cell isolation
**PL-15**	1 week	male	Samples were used for cell isolation and cryopreservation
**PL-16**	1 week	male	Samples were used for cell isolation and cryopreservation

All animals, the wild-type breed, are a mixture of six different breeds: a six-way cross consisting of Yorkshires, Large Whites, Landraces (from the sows), Durocs, Spots, and Pietrans (from the boars). This highly heterogeneous genetic background is desirable in that it parallels the heterogeneous genetic constitutions of human populations.

Supplementary Table 5. Markers of subpopulations in the fetal or neonatal biliary tree, liver and pancreas

	Stem/progenitors	
**Source of cells: Fetal/Neonatal organ**	**Hepato/biliary**	**Pancreas**
**Average size**	9-12 μm	
**Gene expression in the cells in the crypts [Surface antigen expression]**	Oct 4, SOX2, Sall4, BMi-1, SOX17, PDX1<br>$CD45^{-}$, $EpCAM^{-}$, $NCAM^{+}$, $CD44^{+}$,<br>sodium iodide symporter (NIS^{+})	
**Gene expression in cells near the lumens of PBGs. Parallels that found in canals of Hering [Surface antigen expression]**	Pluripotency genes expressed but less so than in cells in the presumptive crypts. Expression of hepatic or biliary tree endodermal transcription factors: SOX9, SOX17, and other hepatic markers (e.g. albumin, AFP)<br><br>[$CD45^{-}$, $EpCAM^{+}$, $CD44^{+}$, ICAM-1^{+}]	No pluripotency gene expression in PDGs in humans; In pigs, it is evident in PDGs near duodenum but not in those distant from duodenum. SOX9, PDX1, NGN3, insulin, amylase<br><br>[$CD45^{-}$, $EpCAM^{+}$, $CD44^{+}$, ICAM-1^{+}]

Supplementary Table 6. Information on Pigs and Cell Numbers				
**Storage#**	**Pigs**	**Cell types****	**Cell number**	**Notes**
S-2	H2B-GFP-Piglet #1 (61105) (1-day-postnatal)	Biliary tree	3,000,000	BTSCs
S-7	H2B-GFP-Piglet #2 (61102) (1-day-postnatal)	Biliary tree	1,000,000	BTSCs
S-11	H2B-GFP-Piglet #3 (61104) (2-day-postnatal)	Biliary tree	1,000,000	BTSCs
S-15	Wild Type-Piglet #1 (1-day-postnatal)	Biliary Tree	570,000	BTSCs
S-20	Wild Type-Piglet #2 (1-day-postnatal)	Biliary Tree	570,000	BTSCs
** gallbladder and extrahepatic biliary tree				

Supplementary Table 7. Quality-control Information for RNA Samples for the sequencing Assays					
**Storage#**	**Cell types****	**RNA Concentration (ng/µL)**	**260/280**	**260/230**	**RIN**
S-2	Biliary tree	102.8	1.95	1.83	5.1
S-7	Biliary Tree	80.3	1.93	1.76	3.8
S-11	Biliary Tree	136.8	1.98	2.09	3.9
S-15	Biliary Tree	340.4	1.97	2.46	n/a
S-20	Biliary Tree	350.7	1.98	2.35	6.4
** Biliary tree cells prepared from gallbladder and extrahepatic biliary tree					

Movies

- **Supplementary Video 1. Porcine intrahepatic and extrahepatic biliary tree.** Post-mortem scan with Iohexol injected into the bile duct for approximately 1 hour. Scan of porcine biliary tree at low magnification to demonstrate the extensive ramifications of the biliary tree from the intrahepatic region to the duodenum.
- **Supplementary Video 2. Common duct of the porcine extrahepatic biliary tree.** Post-mortem scan with Iohexol injected into the bile duct for approximately 1 hour.
- **Supplementary Video 3. Scan of Porcine pancreatic biliary tree.** Four-week-old piglet postmortem scan with Iohexol (6 mls) injected through the accessory pancreatic duct. The tissue was scanned at the time points of 1 min, 5 mins, 10 mins, and 20 mins.
- **Supplementary Video 4. Scan of Porcine pancreatic biliary tree.** Four weeks-old piglet postmortem scan with Iohexol (6 mls) injected through the accessory pancreatic duct; tissue was scanned at the time points of 1 min, 5 mins, 10 mins, and 20 mins.

Animations

- **Supplementary Video 5.** Animation of embryological development of the biliary tree in pigs versus dogs (representative of most mammals)
- **Supplementary Video 6.** Animation of human biliary tree and connections to liver and pancreas.

REFERENCES (Online supplement)

1 Wauthier, E. *et al.* Hepatic stem cells and hepatoblasts: identification, isolation and *ex vivo* maintenance *Methods for Cell Biology (Methods for Stem Cells)* **86**, 137-225 (2008).

2 Wang, Y. *et al.* Biliary Tree Stem Cells, Precursors to Pancreatic Committed Progenitors: Evidence for Life-long Pancreatic Organogenesis *Stem Cells* **31**, 1966-1979 (2013).

3 Schmelzer, E. *et al.* Human hepatic stem cells from fetal and postnatal donors. *Journal of Experimental Medicine* **204**, 1973-1987 (2007).

4 Zhang, W. *et al.* Stem Cell-Fueled Maturational Lineages in Hepatic and Pancreatic Organogenesis. *The Liver: Biology and Pathobiology*, 521-538 (2020).

5. Alvaro, D. and Gaudio, E. Liver capsule: biliary tree stem cell subpopulations. *Hepatology* **64** (2):644. doi: 10.1002/hep.28546. (2016)

6. Cardinale V, et al. Human duodenal submucosal glands contain a defined stem/progenitor subpopulation with liver-specific regenerative potential. *J Hepatol.*78:165-179. doi: 10.1016/j.jhep.2022.08.037. (2023).
