# Supplementary material for: A postnatal network of co-hepato/pancreatic stem/progenitors in the biliary trees of pigs and humans
Source: NPJ Regen Med. 2023 Aug 1;8:40. doi: 10.1038/s41536-023-00303-5 (PMC10394089; doi:10.1038/s41536-023-00303-5)
Supplement: Supplementary file 2 — Reporting summary [file 41536_2023_303_MOESM2_ESM.pdf]

nature portfolio

Corresponding author(s): Lola M. Reid--NPJREGENMED-01062R

Last updated by author(s): 2023/05/22

Reporting Summary

Nature Portfolio wishes to improve the reproducibility of the work that we publish. This form provides structure for consistency and transparency in reporting. For further information on Nature Portfolio policies, see our Editorial Policies and the Editorial Policy Checklist.

Statistics

For all statistical analyses, confirm that the following items are present in the figure legend, table legend, main text, or Methods section.

n/a	Confirmed	
☐	☒	The exact sample size (*n*) for each experimental group/condition, given as a discrete number and unit of measurement
☐	☒	A statement on whether measurements were taken from distinct samples or whether the same sample was measured repeatedly
☐	☒	The statistical test(s) used AND whether they are one- or two-sided<br>*Only common tests should be described solely by name; describe more complex techniques in the Methods section.*
☐	☒	A description of all covariates tested
☐	☒	A description of any assumptions or corrections, such as tests of normality and adjustment for multiple comparisons
☐	☒	A full description of the statistical parameters including central tendency (e.g. means) or other basic estimates (e.g. regression coefficient) AND variation (e.g. standard deviation) or associated estimates of uncertainty (e.g. confidence intervals)
☐	☒	For null hypothesis testing, the test statistic (e.g. *F*, *t*, *r*) with confidence intervals, effect sizes, degrees of freedom and *P* value noted<br>*Give P values as exact values whenever suitable.*
☒	☐	For Bayesian analysis, information on the choice of priors and Markov chain Monte Carlo settings
☐	☒	For hierarchical and complex designs, identification of the appropriate level for tests and full reporting of outcomes
☐	☒	Estimates of effect sizes (e.g. Cohen's *d*, Pearson's *r*), indicating how they were calculated

Our web collection on statistics for biologists contains articles on many of the points above.

Software and code

Policy information about availability of computer code

Data collection	R (version: 4.1.0); sva package (version: 3.42.0); limma package (version: 3.50.1); corrplot package (version: 0.90); pheatmap package (version: 1.0.12); tinyarray package (version: 2.2.6); ggplot2 package (version: 3.3.6)
Data analysis	R (version: 4.1.0); sva package (version: 3.42.0); limma package (version: 3.50.1); corrplot package (version: 0.90); pheatmap package (version: 1.0.12); tinyarray package (version: 2.2.6); ggplot2 package (version: 3.3.6)

For manuscripts utilizing custom algorithms or software that are central to the research but not yet described in published literature, software must be made available to editors and reviewers. We strongly encourage code deposition in a community repository (e.g. GitHub). See the Nature Portfolio guidelines for submitting code & software for further information.

Data

Policy information about availability of data

All manuscripts must include a data availability statement. This statement should provide the following information, where applicable:

- Accession codes, unique identifiers, or web links for publicly available datasets
- A description of any restrictions on data availability
- For clinical datasets or third party data, please ensure that the statement adheres to our policy

The raw data and source code that support the findings of this study are available from the corresponding author upon reasonable request.

Human research participants

Policy information about studies involving human research participants and Sex and Gender in Research.

Reporting on sex and gender	n/a
Population characteristics	n/a
Recruitment	n/a
Ethics oversight	n/a

Note that full information on the approval of the study protocol must also be provided in the manuscript.

Field-specific reporting

Please select the one below that is the best fit for your research. If you are not sure, read the appropriate sections before making your selection.

☒ Life sciences ☐ Behavioural & social sciences ☐ Ecological, evolutionary & environmental sciences

For a reference copy of the document with all sections, see nature.com/documents/nr-reporting-summary-flat.pdf

Life sciences study design

All studies must disclose on these points even when the disclosure is negative.

Sample size	Data were obtained from a minimum of three (or more) biological samples, with at least triplicates of each sample.
Data exclusions	All data generated from the pigs listed in Table S4 (Pigs used for Studies on Anatomy, Histology, Immunohistochemistry or Cell Culture) were included in the manuscript. In the RNA-seq data; we excluded the non-parenchymal cells from the pig liver, to focus only on comparing the similarities of porcine BTSCs with human BTSCs and hHPSCs lineages in order to determine the stemness of porcine BTSCs.
Replication	All experiments were performed at least 3 times, and all conditions were done in three or more separate animals at each time point.
Randomization	Animals used in the study was distributed into anatomy, histology, cell isolating and cell grafting studies at sequential phases. During the patch grafting of the porcine BTSCs into the liver and the pancreas, randomization schedules were provided to the host pigs according to the proposed randomization procedure generated by the nurse and the surgeon at the animal facility.
Blinding	For the patch grafting of porcine BTSCs into the liver versus the pancreas, a double-blinded experimental design was used

Reporting for specific materials, systems and methods

We require information from authors about some types of materials, experimental systems and methods used in many studies. Here, indicate whether each material, system or method listed is relevant to your study. If you are not sure if a list item applies to your research, read the appropriate section before selecting a response.

Materials & experimental systems

n/a	Involved in the study	
☐	☒	Antibodies
☒	☐	Eukaryotic cell lines
☒	☐	Palaeontology and archaeology
☐	☒	Animals and other organisms
☒	☐	Clinical data
☒	☐	Dual use research of concern

Methods

n/a	Involved in the study	
☒	☐	ChIP-seq
☒	☐	Flow cytometry
☒	☐	MRI-based neuroimaging

Antibodies

Antibodies used	Antibodies used are listed in Table S1.
Validation	We have run the validation of each of the antibodies used in the study with positive and negative tissue controls, as well as the negative controls of the experiments, which including the control without the primary or secondary antibodies, repetitively, on the

test tissue, positive tissue and negative controls. The validation was conducted based on the concentrations and staining conditions that are listed in the websites of manufactures.

Animals and other research organisms

Policy information about studies involving animals; ARRIVE guidelines recommended for reporting animal research, and Sex and Gender in Research

Laboratory animals	The pigs used as recipients were a mixture of six different breeds: a six-way cross consisting of Yorkshires, Large Whites, Landraces (from the sows), Durocs, Spots, and Pietrans (from the boars). This highly heterogeneous genetic background is desirable in that it parallels the heterogeneous genetic constitutions of human populations.
Wild animals	n/a
Reporting on sex	n/a
Field-collected samples	n/a
Ethics oversight	All animal experiments were performed in strict accordance with the approved Institutional Animal Care and Use Committees of North Carolina State University (NCSU) in Raleigh and at the University of North Carolina (UNC) in Chapel Hill and so have followed the principles outlined in the Declaration of Helsinki for all human or animal experimental investigations. All procedures used in animal studies and those involving use of human tissues were approved by IACUC and IRB committees at both institutions: UNC at Chapel Hill and at the College of Veterinary Medicine at NCSU. The IACUC approval numbers are 16-316.0 and 17-225.0. Studies for human cells in this project were evaluated by the IRB committee and were provided an IRB approval number of 97-1063.

Note that full information on the approval of the study protocol must also be provided in the manuscript.
